# Supplementary material for: Does perceived scarcity of COVID-19 vaccines increase vaccination willingness? Results of an experimental study with German respondents in times of a national vaccine shortage
Source: PLoS One. 2022 Sep 7;17(9):e0273441. doi: 10.1371/journal.pone.0273441 (PMC9451090; doi:10.1371/journal.pone.0273441)
Supplement: S1 File — (DOCX) [file pone.0273441.s001.docx]

**Supporting Information S1.**

**CONSORT Diagram and Sub-Sample Comparisons**


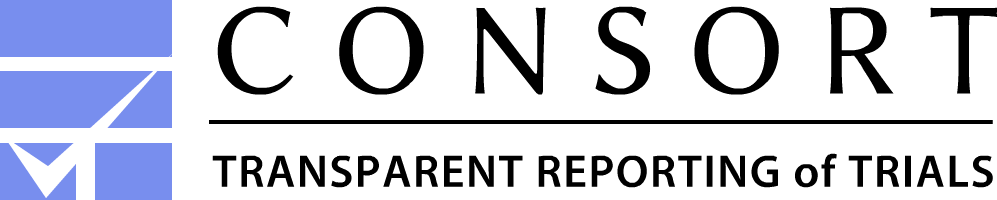


**CONSORT 2010 Flow Diagram**

Randomized (n_6_= 175 )

Assessed for eligibility (n_1_= 238 )

## Enrollment

Excluded (n_2_ = 63 )

♦  Not meeting inclusion criteria:

- RSI > 2 (n = 0)
- Manipulation check failed (n_3_ = 63)
  - Processing time + 2 SD (n_4_ = 16)
  - Processing time - 2 SD (n_5_ = 4)

## Analysis

Surplus condition (n_8_= 77)

Scarcity condition (n_7_= 98)

*Note.* Downloaded from: <http://www.consort-statement.org/downloads>.

**Sample Characteristics of excluded sub-samples**

| Sample  Variable | n_1_ = 238 | n_2_ = 63 | n_3_ = 63 | n_4_ = 16 | n_5_ = 4 |
| --- | --- | --- | --- | --- | --- |
| *Age* | *M* = 35.74  *SD* = 10.26 | *M* = 35.22  *SD* = 11.33 | *M* = 35.22  *SD* = 11.33 | *M* = 38.56  *SD* = 11.79 | *M* = 28.75  *SD* = 2.63 |
| *Gender* | 90 female  148 male | 23 female  40 male | 23 female  40 male | 8 female  8 male | 1 female  3 male |
| *Educational attainment*  completed apprenticeship  baccalaureate  university degree  other secondary school certificate  missing | 18.9 %  28.1 %  44.1 %  7.9 %  1 % | 23.8 %  28.5 %  46.0 %  1.6 %  0.1 % | 23.8 %  28.5 %  46.0 %  1.6 %  0.1 % | 25.0 %  43.8 %  18.8 %  12.4 %  0 % | 25 %  25 %  50 %  0 %  0 % |

*Note*: The participants of n_4_ and n_5_ are also part of n_3_, as they answered incorrectly to the manipulation check variable. n_2_ and n_3_ are thus identical.

**Sample Characteristics of included sub-samples**

| Sample  Variable | n_6_ = 175 | n_7_ = 98 | n_8_ = 77 |
| --- | --- | --- | --- |
| *Age* | *M* = 35.93  *SD* = 9.88 | *M* = 36.02  *SD* = 9.79 | *M* = 35.81  *SD* = 10.05 |
| *Gender* | 67 female  108 male | 36 female  62 male | 31 female  46 male |
| *Educational attainment*  completed apprenticeship  baccalaureate  university degree  other secondary school certificate  missing | 20.6 %  28.0 %  43.4 %  7.4 %  0.6 % | 30.6 %  28.6 %  39.8 %  1.0 %  0 % | 22.1 %  27.3 %  48.1 %  1.3 %  1.3 % |

*Note*: The participants of n_7_ and n_8_ are also part of n_6_.

**Test of Group Differences Between Excluded and Included Participants**

To test whether there is potential bias through the chosen exclusion criteria, it was tested whether the excluded and included sub-samples significantly differed in their sociodemographic composition. An ANOVA with two factors (experimental condition: scarcity versus surplus; filter: included versus excluded) for testing on age differences between the sub-samples revealed no significant difference between the included and excluded participants, *M_included_* = 35.93, *SD* = 9.88, *M_excluded_* = 35.22, *SD* = 11.33, *F*(1, 138) = 0.26, *p* = 607, $\eta^{2}$ = .001. In addition, the interaction between the filter variable and the experimental condition was non-significant, so no age bias was detected between the sub-samples, *F*(1, 138) = 0.09, *p* = 759, $\eta^{2}$ < .001. For gender and educational attainment, two Chi-square tests were conducted to test for group differences. Again, there was no difference between the included and excluded participants with regard to gender, $\chi^{2}$(*N* = 238) = 0.06, *p* = .803, and educational attainment, $\chi^{2}$(*N* = 238) = 3.16, *p* = .788. Also, there was no significant interaction effect between the filter variable and the experimental condition. So, there was no difference between the sub-samples with regard to gender, ${\chi^{2}}_{scarcity}$(*N* = 119) = 0.50, *p* = .477, ${\chi^{2}}_{surplus}$(*N* = 119) < 0.01, *p* = .982, and educational attainment, ${\chi^{2}}_{scarcity}$(*N* = 119) = 1.15, *p* = .949, ${\chi^{2}}_{surplus}$(*N* = 238) = 2.43, *p* = .907.
