# Supplementary material for: Does perceived scarcity of COVID-19 vaccines increase vaccination willingness? Results of an experimental study with German respondents in times of a national vaccine shortage
Source: PLoS One. 2022 Sep 7;17(9):e0273441. doi: 10.1371/journal.pone.0273441 (PMC9451090; doi:10.1371/journal.pone.0273441)
Supplement: S2 File — (DOCX) [file pone.0273441.s002.docx]

**Supporting Information S2.**

**Supplementary Table S2.**

*Results of the multivariate analysis of variance controlling for covariates.*

|  | *Vaccination willingness* | | | *Anger* | | | |
| --- | --- | --- | --- | --- | --- | --- | --- |
|  | *M* | SD | | *M* | | *SD* | |
| *Treatment* |  | |  | |  | |  |
| Scarcity | 4.91 | 1.88 | | 4.75 | | 1.98 | |
| Surplus | 4.31 | 2.07 | | 4.05 | | 2.24 | |
| *F*_treatment_ (1, 174) | 4.22* | | | 4.46* | | | |
| η^2^ | .024 | | | .026 | | | |
| Age | *F*(1, 174) = 1.81  $\eta^{2}$ = .011, *p* = .180 | | | *F*(1, 174) = 0.49  $\eta^{2}$ = .003, *p* = .481 | | | |
| Gender | *F*(1, 174) = 0.02  $\eta^{2}$ < .001, *p* = .897 | | | *F*(1, 174) = 0.23  $\eta^{2}$ = .001, *p* = .629 | | | |
| Education level | *F*(1, 174) = 0.70  $\eta^{2}$ = .004, *p* = .405 | | | *F*(1, 174) = .18  $\eta^{2}$ = .001, *p* = .674 | | | |

*Note*. ^†^*p* $\leq$ .10, ^*^*p* $\leq$.05, ^**^*p* $\leq$ .01, ^***^*p* $\leq$ .001. Treatment effect on the combination of dependent variables:

*F*(2, 168) = 5.80, *p* < .01, η_p_^2^ = .065, Wilk’s $\lambda$ = .935, 1-β = .87.
