# Supplementary material for: Does perceived scarcity of COVID-19 vaccines increase vaccination willingness? Results of an experimental study with German respondents in times of a national vaccine shortage
Source: PLoS One. 2022 Sep 7;17(9):e0273441. doi: 10.1371/journal.pone.0273441 (PMC9451090; doi:10.1371/journal.pone.0273441)
Supplement: S3 File — (DOCX) [file pone.0273441.s003.docx]

**Supporting Information S3.**

**Supplementary Table S3**

*Results of the multivariate analysis of variance on the single items.*

|  | *VW_1* | | *VW_2* | | | *VW_3* | | *Anger* | | | |
| --- | --- | --- | --- | --- | --- | --- | --- | --- | --- | --- | --- |
|  | *M* | SD | *M* | | *SD* | *M* | *SD* | *M* | | | *SD* |
| Scarcity | 5.43 | 2.01 | 4.42 | | 2.07 | 4.89 | 2.03 | 4.75 | | | 1.98 |
| Surplus | 4.89 | 2.25 | 4.13 | | 2.12 | 4.00 | 2.10 | 4.04 | | | 2.24 |
| *F*_treatment_ (1, 172) | 2.74^†^ | | | 0.82 | | 7.89** | | |  | 4.88* | |
| η^2^ | .016 | | | .005 | | .044 | | |  | .028 | |

*Note*. ^†^*p* $\leq$ .10, ^*^*p* $\leq$.05, ^**^*p* $\leq$ .01, ^***^*p* $\leq$ .001. Treatment effect on the combination of dependent variables:

*F*(4, 168) = 5.60, *p* < .01, η_p_^2^ = .118, Wilk’s $\lambda$ = .882, 1-β = .98

VW_1: “Will you get vaccinated as soon as possible?” (1 = very unlikely, 7 = very likely)

VW_2: “I will be trying harder to get a vaccination appointment.” (1 = very unlikely, 7 = very likely)

VW_3: “If necessary, I will use different channels (e.g., primary physician and vaccination center) to obtain a vaccination appointment.” (1 = very unlikely, 7 = very likely)

Anger: “I am angry about the current debate on advantages and liberties for vaccinated people.” (1 = completely disagree, 7 = completely agree)
