# Supplementary material for: Does perceived scarcity of COVID-19 vaccines increase vaccination willingness? Results of an experimental study with German respondents in times of a national vaccine shortage
Source: PLoS One. 2022 Sep 7;17(9):e0273441. doi: 10.1371/journal.pone.0273441 (PMC9451090; doi:10.1371/journal.pone.0273441)
Supplement: S4 File — (DOCX) [file pone.0273441.s004.docx]

**Supporting Information S4.**

**Supplementary Table S4**

*Results of the multivariate analysis of variance weighted by participants’ gender.*

|  | *Vaccination willingness* | | | *Anger* | | | |
| --- | --- | --- | --- | --- | --- | --- | --- |
|  | *M* | SD | | *M* | | *SD* | |
| *Treatment* |  | |  | |  | |  |
| Scarcity | 4.91 | 1.88 | | 4.75 | | 1.98 | |
| Surplus | 4.31 | 2.07 | | 4.05 | | 2.24 | |
| *F*_treatment_ (1, 174) | 4.03* | | | 4.77* | | | |
| η^2^ | .023 | | | .027 | | | |

*Note*. ^†^*p* $\leq$ .10, ^*^*p* $\leq$.05, ^**^*p* $\leq$ .01, ^***^*p* $\leq$ .001. Treatment effect on the combination of dependent variables:

*F*(2, 168) = 5.91, *p* < .01, η_p_^2^ = .065, Wilk’s $\lambda$ = .935, 1-β = .87. The gender weight factors were 1.39 for female participants and 0.76 for male participants accounting for a higher representation of women among the unvaccinated population (adapted from Coscia, 2021).

**References**

Coscia, Verena (October 6, 2021). *Vaccination willingness in Europe: Who are the unvaccinated?* Max Planck Institute for Social Law and Social Policy. Retrieved from <https://www.mpisoc.mpg.de/en/newsroom/news/detail/announce/vaccination-willingness-in-europe-who-are-the-unvaccinated/>
