# Supplementary material for: Does perceived scarcity of COVID-19 vaccines increase vaccination willingness? Results of an experimental study with German respondents in times of a national vaccine shortage
Source: PLoS One. 2022 Sep 7;17(9):e0273441. doi: 10.1371/journal.pone.0273441 (PMC9451090; doi:10.1371/journal.pone.0273441)
Supplement: Attachment — Submitted filename: PONE-D-21-30612_review.docx [file pone.0273441.s005.docx]

Summary

The study explores the association between the perceived scarcity of the COVID-19 vaccine and people’s willingness to get vaccinated. The topic is very timely and is of significant importance. However, there are a number of issues that need to clarified about the study design to ensure its validity and about its limitations and the effect of these limitations on the results and their interpretation. To account for potential confounders and quantify the effect size accurately, I strongly recommend Author perform multivariate regression analysis, instead of one-way MANOVA. If this cannot be done, Author should explain the reasons in the discussion as it seems to be a better study design to examine the association between perceived scarcity of vaccines and willingness to get vaccinated. To be acceptable for publication, the manuscript requires substantial revision as follows.

Introduction

Author described in detail the impact of vaccine scarcity on consumer demand and its applicability to the current context of COVID-19 vaccine roll-out. In general, the introduction described the current situation in Germany well to provide context to the study. However, all these factors mentioned in the introduction should be accounted for in the analysis. Also, there is little justification for the reason why Author decided to measure the “anger” as an exploratory analysis (i.e., why is it of importance and what is its relevant to the main hypothesis?).

Page 3, paragraph starting with “As for the context of vaccines, …”: This sentence does not appear to have the correct citation. Pls check and include an appropriate citation.

Page 4, sentence starting with “At first, those countries…” requires citation.

Page 4, sentence starting with “However, despite all these initial obstacles, …”: When you say “the period of the national vaccine shortage”, please be explicit and state the exact period in the text.

Page 5, paragraph starting with “In addition to the test of a scarcity…”: This is the first time Author introduced the debate over relaxation of measures for those vaccinated. If this is one of the outcome measures you aim to test in your study, please provide more context as to its importance before introducing a question related to it.

Methods

Fundamentally, I am concerned with the validity of the study design.

1) Author should not describe this study as an experimental study. There is no counterfactual (pre-/post-test or treatment vs. control) group or randomization. The study appears to be a cross-sectional study.
2) The ‘treatment’ described in the study is not a true treatment. The instant exposure to crafted information on vaccine surplus/scarcity is unlikely to lead to an immediate change in participants’ perception about vaccination. Therefore, exposure to treatment -> associated outcome relationship here is not established.
3) Similarly, the study design does not rule out the possibility that the participants’ willingness to be vaccinated was established prior to their exposure to information on vaccine surplus/scarcity. There is no pre-/post- comparison to establish the temporal relationship between the exposure and the outcome. The current results without this does not support a causal relationship.
4) In addition, Author used one-way MANOVA to test the hypothesis, which does not account for the potential confounding effects. This way, it cannot be ruled out that the participants’ willingness to be vaccinated is driven/mediated by other factors than the perceived scarcity of vaccines. This contradicts what was stated in the introduction and the discussion that vaccine efficacy or side effects also can drive people’s willingness to be vaccinated. Author needs to justify why one-way MANOVA was used on the set of outcome variables. Author should also separately examine and present in a table whether there are any groups differences with regard to demographic and other confounding variables to bolster the findings.
5) Lastly, it is unclear why Author did not choose to perform multivariate regression analysis on each of the outcome variables to account for potential confounding factors. This should be explained in the discussion.

Currently, your methods section is a lengthy walk-through of the online survey. Some information you included, such as how participants were “thanked for their participation”, is not essential. Rather than describing the survey procedure in detail, please provide the well-articulated summary of what you were trying to measure, how they were measured in the survey (i.e. type of question or scale used), and how they were coded in the data. Also, whenever a technical term is introduced, spell it out the first time it was used, and explain what it is used for.

Page 5, sentence starting with “Invitation mails were sent out…”: What was the sampling pool for this invitation? what is meant by “weighted by” age and gender?

Page 6, paragraph starting with “Participants then answered questions…”: What are the dependent variables and what do you mean by ‘manipulation check’? What do you mean when participants were not directed to the “participation code”? What is the “debriefing information”? Are these important facts to be mentioned in this section?

Page 7, “Dependent variables”:

1) What was your rationale to include these three questions to measure the outcomes? Are these questions from a validated instrument to measure willingness to receive vaccination? (If yes, please cite the work.)
2) Why is the scale from 1 – 7? It appears to be unconventional.
3) Did you combine the three questions to form one outcome measure scale? If so, how did you combine them? (i.e., average of 3, sum of 3, etc.).
4) What is the ‘alpha’? (if it is Cronbach’s alpha, you need to specify it explicitly in the text and explain what it means)

Page 7, “Manipulation check”:

What is the purpose of measuring this variable?

Page 7, “Sample size and data analysis”

1) In the first sentence, please clarify what a “large effect size” is and a “small effect size” is. Please provide appropriate ranges.
2) For the power calculation, please specify d, 1-beta, alpha. Also, please spell-out MANOVA when it appears for the first time in the text.
3) In the last sentence, what do you mean when you say “A one-way MANOVA …. was pre-registered?”

Page 7, “Exclusion criteria”

1) What is a relative speed index? Please describe.
2) Spell out SD when it appears for the first time in the text.
3) The description of the final sample for the study should be provided in the “Results” section using a table including all the variables used in the analysis. Please provide such a table in this section.

Results

1) Currently the results section lacks a descriptive table of the sample (a conventional table).
2) In addition, the way in which the results is reported in the text is hard to follow. A table for the results of the one-way MANOVA analysis for each group should be included. Author listed a list of abbreviated terms with figures without explaining what they mean. There is no effect size reported for the treatment, or no justification why the different tests were used to report the results.

Discussion

Overall, your discussion is based on the findings from your study to draw general inference on the German population. I am concerned on the validity of the discussion in general because 1) the generalizability of the findings based on a small sample of 175 participants is not discussed; and 2) the current study design does not allow any causal inference. Limitations should also expand further to accommodate the concerns raised in the “Methods” section.

Page 8, sentence starting with “The results support the hypothesis…”: Based on the current study design, there is very little evidence generated from the study to support this statement.

Page 9, sentence starting with “Surprisingly, this has not..”: You cannot say this without testing for this. Your current study does not explore the association between the mentioned factors and your dependent variable.

Page 9, sentence starting with “In fact, when some cities…”: This is irrelevant to the findings of this study and should rather presented in the introduction.

Page 9, sentence starting with “On the other hand, scarcity mentalities..”: This sentence is vague and does not add any value to the discussion as it is formed. Author should clarify it.

Page 9, sentence starting with ”Such anger may also quickly…”: Is there any literature documenting such an association? If yes, Author should cite it. This could be an interesting mechanism and Author should clearly state this in the introduction to justify the exploratory question (focusing on measurement of anger).

Minor revisions:

No lines numbers provided in the submission file. This made the review of this manuscript difficult which will also make its revision similarly difficult.

Referencing style does not comply with PLOS ONE’s submission guideline, and in-text citation format is inconsistent throughout (i.e., avoid adding hyperlink to a webpage directly in the text, use a citation software so that in-text citations are numbered in Vancouver style).

Footnotes are not permitted as per PLOS One’s guidelines.

Overall, writing needs to be improved in terms of grammar and style. For example, inconsistent use of tense throughout the text; inconsistent use of terminology on “COVID-19 vaccine” (vs. SARS-CoV-2 vaccine)
