# Supplementary material for: Does perceived scarcity of COVID-19 vaccines increase vaccination willingness? Results of an experimental study with German respondents in times of a national vaccine shortage
Source: PLoS One. 2022 Sep 7;17(9):e0273441. doi: 10.1371/journal.pone.0273441 (PMC9451090; doi:10.1371/journal.pone.0273441)
Supplement: Attachment — Submitted filename: Response_Letter_PONE.docx [file pone.0273441.s006.docx]

Dear Dr. Delcea,

I would like to resubmit the revised version of the manuscript entitled “Does perceived scarcity of COVID-19 vaccines increase vaccination willingness? Results of an experimental study with German respondents in times of a national vaccine shortage.” (PONE-D-21-30612)

I would like to thank you and the reviewers for your assessment and evaluation of the manuscript!

I made some major revisions which I shortly want to outline here. First, I have added additional information in the supplements regarding the sample characteristics of the excluded participants by adding a CONSORT diagram as asked for by reviewer 1. Second, I have added additional analyses on the single items as raised by reviewer 4 mainly showing the same pattern of results. Third, I have added an additional test of whether the sample is balanced regarding sociodemographic information which is the case. Also, I added an additional t-test on whether the manipulation check has worked across the whole sample – which also was the case. Last, according to concerns raised by reviewer 4 and 6, I have lowered the tone in the discussion a bit so that the results of the study do not seem to be overestimated.

I have tried to implement all addressable points in the revision and used clearer language in many parts of the manuscript to overcome possible misunderstandings. With regard to some questions on the experimental method or, for example, the question of what a manipulation check is, which has been raised in some of the reviews, I have attempted to clear them by giving brief answers in my response letter.

Overall, I want to highlight that this is a **short report** on the results of a small preregistered study on scarcity effects in the context of the COVID-19 pandemic. As such one, to my experience and opinion, it has several strengths which I am not used to typically see in this category of research articles. First, the study has been preregistered to the data collection and and the results support the preregistered hypothesis. Second, the preregistration, data, and syntax have been immediately made public after data collection in order to meet the open science criteria. Third, the short report deals with a (still) highly topical issue, as a next wave of vaccination shortage can be expected when the omicron-specific SARS-CoV-2 vaccines will be available.

Attached is a revised version of the manuscript with tracked changes and an untracked version.

In this response letter, I have bolded my responses to the reviewers’ comments.

Thank you for giving me the chance to having revised and resubmitted the manuscript!

Best,

The author

_____________________________________________________________________________________________________

5. Review Comments to the Author

Please use the space provided to explain your answers to the questions above. You may also include additional comments for the author, including concerns about dual publication, research ethics, or publication ethics. (Please upload your review as an attachment if it exceeds 20,000 characters)

Reviewer #1: This is a well written and concise paper reporting a useful survey of the impact of Covid vaccine scarcity on the likelihood of unvaccinated individuals to get vaccinated.

The author should detail the number of participants that were excluded according to each of the exclusion criteria. This could either be in the text or in a CONSORT-type diagram.

**I thank reviewer 1 for their overall evaluation of the manuscript and I agree that a CONSORT diagram is useful given the exclusion of respondents who failed the manipulation check. This is why the sample description was revised and a CONSORT Flow Chart was added in the online supplements (l. 160-174):

“The exact sample sizes of the excluded sub-groups as well as their characteristics are reported in a CONSORT flow diagram in the online supplement (S1). The final sample size consisted of 175 participants (108 male, 67 female; Mage = 35.93, SD = 9.88; education level: 43.4% university degree, 28% baccalaureate, 20.6% completed apprenticeship; 7.4% other secondary school certificate). The sample is not representative for the entire German population. But, this was not the intention of the study. More importantly, the sample was intended to correspond to the characteristics of the unvaccinated population at the time of the survey. According to the German Federal Statistical Office, the average age of the sample under research roughly corresponds to that of the unvaccinated population in May 2021 (Statistisches Bundesamt, 2021). There is no available data for the distribution of gender and educational attainment among non-vaccinated individuals in Germany, at the field time of the survey (see Impfdashboard of the Robert-Koch-Institute, 2022). However, later data on vaccination intentions has shown that women tend to be rather unvaccinated than men (e.g., Graeber et al., 2021), indicating a potential over-representation of male respondents in the sample under research.”**

Page 4: ‘especially Germany’ would read better as ‘Germany especially’

**Thanks for this language advice! It has been changed in the revised manuscript.**

Top of page 6: ‘have a fix vaccination appointment’ - ‘fix’ should be replaced with ‘fixed’

**Again, thanks a lot, I changed it.**

Page 6, near the bottom’: Since deception took place, participants were not directed to the participation code until they indicated that they had read and understood the debriefing information.’ - it’s not clear what the ‘participation code’ is.

**I agree that this first read a bit unspecific. I revised it in the new version of the manuscript (l. 119-123):**

**“Since deception took place, participants were not directed to the last survey page including the participation code for the payment until they indicated that they had read and understood the debriefing information. This has been implemented for ethical reasons, so that the participants would be informed about the true purpose of the study in any case.”**

Top of page 7: the author should define the alpha that is given here.
Sample size calculation: please define the parameters.
Results: please define the parameters (M, SD, etc).

**I agree with reviewer 1 and added the additional information (l. 127-143):**

***“Dependent variables*. Participants’ vaccination willingness was measured the mean value of the following three items: “Will you get vaccinated as soon as possible?”; “I will be trying harder to get a vaccination appointment in the near future.”; “If necessary, I will use various channels (e.g., primary physician and vaccination centers) to get a vaccination appointment.”; reaching from 1 = “very unlikely” to 7 = “very likely”, Cronbach’s α = .93, *M* = 4.65, *SD* = 1.98. The naming of the dependent variables differs from the preregistration. In the preregistration, vaccination willingness and appointment intention were mentioned separately. For reasons of conceptual overlap and high internal consistency, the items were combined into one scale.**

**For the explorative analysis, participants were also asked to answer whether they are angered by the debate over relaxations for vaccinated individuals (“I am angry about the current debate over advantages and liberties for vaccinated people,” 1 = “completely disagree” to 7 = “completely agree”, *M* = 4.44, *SD* = 2.12).**

***Manipulation check*. Perceived vaccine availability was measured with a single item: “How do you feel about the supply situation for COVID-19 vaccines in your county?” ranging from 1 = “very poor” to 7 = “very good”, *M_overall_* = 3.66, *SD* = 1.27.”**

**The information about the sample size estimation was changed as follows (l. 147-151):**

**“An a-priori power analysis using G*Power (version 3.1, Faul et al., 2009) for the detection of a medium to large scarcity effect in a Multivariate Analysis of Variance (MANOVA) including two dependent variables with *d* = .60, 1-β = .95, α = .05 resulted in a required sample size of 158 participants. To allow for potential dropout, data from 238 individuals was collected.”**

Has the author assessed the validity of the assumptions underlying MANOVA, eg. Normality of the response? It seems likely to me that they will not hold and that a nonparametric alternative may be necessary.

**Yes, the assumptions for conducting a MANOVA have been assessed. Homogeneity of variances within both treatment conditions was given, whereas the distribution on the response variables differed somewhat from normal distribution. I want to argue that there are several reasons why it is plausible that the dependent variables deviate from normal distribution. First, since the beginning of the pandemic, vaccination has been a very polarized issue resulting in many people being either pro or contra COVID-19 vaccines, which is also reflected by the data. Second, at the time of the survey, there was more uncertainty about the existing vaccines, their safety, and possible side effects, which may have also influenced the distribution of the dependent variables. However, the (M)ANOVA is known to be a statistical tool which tends to be especially robust towards violations of the normal distribution assumption (Blanca et al., 2017; Schmider et al., 2010).**

**Nevertheless, the analysis was repeated with box-cox transformed dependent variables yielding the same pattern of results as reported in the paper – with and without the inclusion of covariates (**[**https://osf.io/pzury/?view_only=7ebaf5889d3b49cb8e5448c5f2f88b21**](https://osf.io/pzury/?view_only=7ebaf5889d3b49cb8e5448c5f2f88b21)**).**

The author should provide some interpretation and discussion of the magnitude of the differences between the groups. The difference in means was less than 1 between the groups for both outcomes and I would argue that this actually a very small difference - perhaps even to the extent that this is a positive thing and may mean that people are relatively likely (both groups >4) to get vaccinated regardless of the availability of the vaccine. I think there is a nuance between the statistical and societal significance of the results that has been missed by the author.

**The effect size was mentioned referring to the range of small effect sizes (l. 217-219):**

**“Results showed a small but significant scarcity effect on both dependent variables (with both effect sizes .010 < η^2^ < .039 indicating a small effect, see Lenhard & Lenhard, 2016).”**

**Also the discussion was revised in order to relativize the findings in accordance with the effect sizes observed (l. 284-290):**

**“(…) This has left the impression that the scarcity of SARS-CoV-2 vaccines may increase the willingness of individuals to be vaccinated. However, this observation at the macro level did not allow for any causal conclusions on the micro level of vaccination behavior (Ramström, 2018). For this reason, an experimental study was conducted to test whether a scarcity impression of the SARS-CoV-2 vaccine can indeed lead to an increased willingness to vaccinate.**

**The results of the presented study support the idea that perceived scarcity of SARS-CoV-2 vaccines may be *one* driving factor for increasing people’s vaccination willingness. Of course, only limited conclusions about the complexity of vaccination decisions can be drawn from such a small study, and a cross-sectional experiment like this one only represents a momentary snapshot, through which it is not possible to construct a comprehensive explanation of the processes of the last weeks and months.**

**That said, the results of this study do fit into a larger picture of scarcity effects in varying fields and confirm that scarcity information has a promoting effect on the demand of a good (Aggarwal et al., 2011; De Janvry et al., 2006; Gierl & Huettl, 2010; Jang et al., 2015). Another question is whether such effects are desired in the vaccination context. On the one hand, in the fight against the COVID-19 pandemic, it is highly desirable to achieve herd immunity as quickly as possible by maximizing vaccination rates. On the other hand, scarcity effects represent a rather superficial form of information processing (see, e.g., Mittone & Savadori, 2009; Verhallen, 1982). In this vein, Pereira and colleagues (2021) found that perceived scarcity of SARS-CoV-2 vaccines increased vaccination willingness among people with low compassion for others, but decreased it among those with high compassion for others. Scarcity induced vaccination willingness may disappear the moment vaccines become widely available. From an informed patient perspective, it would therefore be more desirable to increase vaccination willingness through deeper persuasion (see, e.g., Argote et al., 2021; Gowda et al., 2013).”**

Figure 1 should be displayed as box plots instead of the bar plots shown. This would help with the interpretation of the results, as referred to in the previous point, since it will give an overview of the entirety of each group.

**Again, thanks for this advice! Figure 1 has been replaced by a boxplot.**

Reviewer #2: -the author should be removed "see e.g." from the citations (see e.g. , Westdeutscher Rundfunk, May 2021).
-the author should be included the list of abbreviations eg. WHAT does MANOVA, IBM, SPSS and COVID-19 means?

**I think for most researchers in the field these are frequently used abbreviations which are typically used in scientific papers. According to APA guidelines, it is absolutely standard to abbreviate the data analysis software, such as SPSS or R.**
**Nevertheless, I changed it in the revised version of the manuscript (l. 147-150, l. 179-180):**

**“An a-priori power analysis using G*Power (version 3.1, Faul et al., 2009) for the detection of a medium to large scarcity effect in a Multivariate Analysis of Variance (MANOVA) including two dependent variables with *d* = .60,** **1-β = .95, α = .05 resulted in a required sample size of 158 participants.”**

**“The IBM Statistical Package for the Social Sciences (SPSS) 26 was used for the statistical analyses.”**

-write the formula and assumption of MANOVA?

**A MANOVA is a standard model (not only) in psychological research providing a general test of whether the variance of certain response variables differs to a higher extent between experimental conditions than within experimental conditions. It is very unusual (not recommended by the reporting guidelines of the field) to precise this by any formula. I know, this may be more the case in fields of economic modelling or when using regression models in other disciplines, but it is typically not done when using a standard model of comparing two experimental conditions with each other.**

**See Nyhus Hagum et al. (2022), Roznik & Alford (2015), or Tricarico et al. (2011) for some examples of PLOS one articles using a MANOVA without specifying the underlying mathematical formular (which would be also odd for the underlying study).**

- the methods of this study like lecture notes so rewrite again

**Also in line with some comments raised by reviewer 4, I revised many parts of the methods section of the paper (l. 119-177).**

Reviewer #3: Dear author,
The abstract missing the conclusion part, there are still characters left to add that part.

**Thank you for this advice! I have followed reviewer 3’s recommendation and added a conclusion part to the abstract.**

Was validity of the survey assessed?

**The validity of the survey is mainly expressed by the internal consistency of the scales used in the questionnaire. These were on an extremely satisfying level (Cronbach’s alpha > .80, see Cortina, 1993).**

Many of the references used here are quiet old, additionally, it is unreasonable to compare the scarcity of condiments to that of medical procedures.

**Again, thanks for the recommendation! I have added more recent references (also in the context of COVID-19 vaccines).**

**e.g.:**

**Hajek, K. V., & Häfner, M. (2021). Paradoxes of Reactance During the COVID-19 Pandemic: A Social-psychological Perspective. Javnost-The Public, 28(3), 290-305. https://doi.org/10.1080/13183222.2021.1969619**

**Pereira, B., Fehl, A. G., Finkelstein, S. R., Jiga‐Boy, G. M., & Caserotti, M. (2021). Scarcity in COVID‐19 vaccine supplies reduces perceived vaccination priority and increases vaccine hesitancy. Psychology & Marketing. https://doi.org/10.1002/mar.21629**

**Sprengholz, P., Betsch, C., & Böhm, R. (2021). Reactance revisited: Consequences of mandatory and scarce vaccination in the case of COVID‐19. Applied Psychology: Health and Well‐Being, 13(4), 986-995. https://doi.org/10.1111/aphw.12285**

**However, generally spoken, I would nevertheless argue that, first, research is not like cheese that is going bad with time (an analogy which I was taught by my supervisor), second, the psychological processes that lead people to find scarce goods more attractive may be the same across different classes of products and are likely to be the same regardless of the time in which people live.**

Reviewer #4: This short paper experimentally tests whether information about COVID-19 vaccine scarcity increases vaccine willingness in Germany in May 2021. Participants were recruited online for a short survey their manipulated (with deception) whether respondents were informed that their county was or was not experiencing vaccine shortages, before assessing the impact of this treatment on immediate self-reported willingness to vaccinate as well as anger about restrictions on the unvaccinated. The results show about a 0.3 standard deviation increase in willingness to vaccinate, suggesting – in line with studies relating to non-COVID vaccines – that scarcity can increase willingness to get vaccinated.

While the paper is concise and speaks broadly to a key topic – determinants of vaccine uptake – there are also substantial limitations in terms of what can be learned from this study. As noted in detail below, the writeup is missing important information, there are reasons to doubt the value of the findings, and the theoretical mechanisms are not explored.

The concision of the paper is good, but it goes too far. It is actually quite difficult to evaluate the study due to a lack of standard information about the study. In particular:

• None of the assumptions required for valid inference are supported empirically. There are no tests validating that treatment is balanced across predetermined covariates (location and demographic variables, in this case), which is important in studies like this with a small sample. Moreover, there are no tests of differential attrition – do people exposed to different messages answer the main outcome questions at different rates? If so, is the sample that survives to the outcome questions still well balanced?

**I thank reviewer 4 for their advice! It is true, that (although conducted) there was no report of a systematic test between the both treatment groups. This was added with regard to the relevant covariates (l. 174-183):**

**“Prior to the main analysis, I tested whether the experimental conditions varied significantly regarding the distribution of sociodemographic characteristics to control for possible biases. Overall, *n* = 98 respondents were assigned to the scarcity condition and *n* = 77 to the surplus condition. The sub-samples did not significantly differ regarding participants’ age, *M*_age scarcity_ = 36.02, *SD* = 9.80, *M*_age surplus_ = 35.81, *SD* = 10.05, *t*(173) = 0.14, *p* = .887, nor gender, χ^2^ (1, *N* = 175) = 0.23, *p* = .642, nor educational attainment, χ^2^ (6, *N* = 175) = 3.18, *p* = .786. With this result, systematic biases between treatment conditions can be excluded, which allows potential treatment effects to be attributed to the experimental manipulation.”**

**I did not analyze the dropout rates of the entire participation, because the Survey Platform defaults to downloading only complete data sets and to directing only participants who have completed the questionnaire to the final participation code. However, in this study I don't see any theoretical benefit of analyzing the dropout rates either. I find it more important that the final treatment groups are approximately equal in size and balanced with respect to sociodemographic variables. This is the case as described above.**

• The author notes that survey invitations were sent in order to match the age and gender population distribution. However, this does not tell us what the sample that actually takes the survey looks like – is it representative on these dimensions or any other dimensions that were not matched on? Ultimately, we know very little about the sample, making it hard to evaluate whether this online sample is representative of the German population (or other populations that we might want to extrapolate to).

**Again, I agree with reviewer 4’s concerns and added further information on the representativity of the sample under research. In this context, it is important to note that the aim of the study was not to investigate scarcity effects among a sample which was representative for the general population, but among a sample which was roughly representative for the population of *non-vaccinated* individuals at the time of the study. Therefore, the following section was added (l. 162-172):**

**“The sample is not representative for the entire German population. But, this was not the intention of the study. More importantly, the sample was intended to correspond to the characteristics of the unvaccinated population at the time of the survey. According to the German Federal Statistical Office, the average age of the sample under research roughly corresponds to that of the unvaccinated population in May 2021 (Statistisches Bundesamt, 2021). There is no available data for the distribution of gender and educational attainment among non-vaccinated individuals in Germany, at the field time of the survey (see Impfdashboard of the Robert-Koch-Institute, 2022). However, later data on vaccination intentions has shown that women tend to be rather unvaccinated than men (Graeber et al., 2021), indicating a potential over-representation of male respondents in the sample under research.”**

• A manipulation check was conducted, which is good, but the results of it are not reported. Did the manipulation work, and for how many people did it work? This is important because it’s hard to gauge whether the intensity of treatment is – i.e. how many people changed their minds because of treatment. This also has implications for whether the mechanism driving the results is belief updating or increasing salience.

**I agree with reviewer 4’s comment. I added information on the t-test comparing both treatment groups on the MC variable (l. 192-195):**

**“In addition, results of a t-test between the scarcity and surplus condition regarding the manipulation check variable revealed that individuals in the scarcity condition perceived the supply situation as significantly worse than those in the surplus condition, *M*_scarcity_ = 2.93, *SD* = 1.10, *M*_surplus_ = 4.58, *SD* = 0.78, *t*(172) = -11.14, *p* < .001.”**

**Please note that, although not reported in the paper, this difference on the MC variable kept robust when no exclusion criteria have been used:**

***M*_scarcity_ = 3.37, *SD* = 1.41, *M*_surplus_ = 3.81, *SD* = 1.32, *t*(234) = -2.43, *p* = .016**

• The main vaccination intention outcome is comprised of 3 survey questions. However, the author does not report how the 3 items are combined – is the main outcome the average of the 3, an inverse-covariance weighted scale, a factor, or something else? In addition, it would be nice to see the results reported by item separately, since the second and especially the first outcomes seem to be the most relevant ones for understanding vaccination intentions, but may not be the outcomes that are driving treatment effects.

**Thanks for this advice! I also added a separate MANOVA on the single items in the supplements which mainly show the same pattern of results (S3).**

• It is also worth noting that the preregistration information deviates somewhat from the final paper. The sample size differs, another outcome is introduced in the paper, the scale for vaccination outcomes is not mention in the analysis plan, the definition of treatment failing was not defined in advance, etc. These are not major issues, but some justification for deviation should be provided.

**Yes, I agree with reviewer 4’s comment on the deviation from the preregistration. This was now made more clear in the revised version of the manuscript, e.g.:**

**“The naming of the dependent variables differs from the preregistration. In the preregistration, vaccination willingness and appointment intention were mentioned separately. For reasons of conceptual overlap and high internal consistency, the items were combined into one general vaccination willingness scale.”**

**“This item was added for explorative reasons, but was not part of the preregistration.”**

**“This is why a minimum sample size of 120 participants was mentioned in the preregistration. In addition, an a-priori power analysis using G*Power (version 3.1, Faul et al., 2009) for the detection of a medium (*d* = .50 to .70) to large (*d* = .80 to** $\boldsymbol{\geq}$ **1, Lenhardt & Lenhardt, 2016) scarcity effect in a Multivariate Analysis of Variance (MANOVA) including two dependent variables with *d* = .60, 1-β = .95, α = .05 resulted in a required sample size of 158 participants. To allow for potential dropout, data from 238 individuals was collected.”**

**But, excluding participants for whom the treatment has not worked has been previously preregistered as an exclusion criterion: “participants for whom the treatment failed“. (**[**https://aspredicted.org/blind.php?x=kt8us2**](https://aspredicted.org/blind.php?x=kt8us2)**)**

Despite the experimental nature of the study, I have several concerns about the design itself:
• First, the outcomes are vaccine intentions measured immediately after a manipulation that respondents were told was being done to them. This raises important concerns about social desirability biases. Did people thinking vaccines were scarce feel the need to answer that they will get one, out of shame or experimenter demand? To be fair, most studies in the literature have used survey intentions – rather than behavioral outcomes – to measure vaccination, but this is increasingly unsatisfactory given that vaccines are now widely available (and were fairly available in Germany in May 2021 too) and because this particular survey started by telling participants “that they might be deceived during participation and that the true purpose of the study could not be explained until the end.”

**I understand reviewer 4’s concerns. But, I have to disagree a little bit regarding the statement that COVID-19 vaccines have been fairly available in May 2021 in Germany. The effective vaccination rate has been around 40 % at the time of the experiment (see** [**https://impfdashboard.de/en/**](https://impfdashboard.de/en/)**) and COVID-19 vaccines have been scarce (see e.g., Warren & Lofstedt, 2021 for a discussion). In addition, many citizens were disappointed by the vaccination campaign of the German government regarding the high bureaucratic burden and ongoing supply problems.**

**The advantage of the treatment, however, at this time, was that people could not really assess whether their county would be one of those with many vaccine doses or few vaccine doses in the future. Therefore, the treatment tied into this point and the scarcity impression was manipulated by saying that the participant’s county would be either among the poorly supplied (scarcity) or among the well supplied (surplus).**

**With regard to informed consent, according to the local ethics commission of my university, it must always include information that participants may be deceived if deception is involved in the study. However, the informed consent form is very long and the question arises whether it is fully recalled by participants or whether the information about deception is perceived as one of many other pieces of the informed consent, and thus may not have been recalled by them. In addition, the manipulation check variable guaranteed that only participants were included in the final data analysis who believed in the presented information. This is the strongest guarantee that can be provided in order to avoid pure desirability effects (Fiedler et al., 2021). Also, the manipulation check was measured after the assessment of the dependent variables which also excludes the risk that this measure biases the results in a certain direction (Kidd, 1976).**

• Second, the exclusion criteria seem to risk post-treatment bias. While excluding “speeders” is fine, I worry about excluding people on the basis of their posterior beliefs about scarcity. It is unorthodox in my field to condition the sample (or use covariate adjustment) on basis of post-treatment covariate, as this introduces biases, see e.g. Hernan and Robins 2011. I would encourage the author not to use this potentially bias-inducing exclusion restriction. If they wish to normalize by the degree to which the manipulation works, they would be better served using an instrumental variables regression. This should be easy to implement in a revision, although it’s not clear how robust the results would be.

**At least in social psychological research it is common practice to define exclusion criteria for extremely short *and* long processing times. Both are related to low data quality (see Leiner, 2019 for a discussion). Relatedly, Draisma and Dijkstra (2004) found that longer latencies are related to higher response error rates. However, there were no additional participants being excluded by the speed variables – they were already part of the subsample excluded by the failed manipulation. So there is no additional bias by this exclusion criteria (nevertheless, I mentioned them because they were part of the preregistration).**

• Third, it wasn’t clear to me whether the treatment would be interpreted by respondents as reflecting low supply of or high demand for vaccines. The former sentence in the treatment suggests a supply mechanism, but the latter could be either. This has implications for interpreting the results.

**The scarcity impression was mainly manipulated by telling participants that their county will face a below-average supply. The experimental manipulation read as follows:**

**“According to a press release from the Department of Health, your county is expected to receive significantly fewer [more] doses of the COVID-19 vaccine than other counties will in the coming weeks. Consequently, a particularly high shortage [availability] of vaccines is expected in your region.“**

Finally, I had several concerns regarding the broader contribution:
• The experiment has very little to say about what theoretical mechanisms might be driving the scarcity effect. I think a variety of mechanisms, with differing policy implications, could be at play – is scarcity altering perceptions of the value of vaccination, due to limited number or social learning (i.e. high uptake suggests others think vaccines are good)? Is scarcity creating pressures to conform because people are learning that others around them are getting vaccinated? Is scarcity simply galvanizing people into action, but without altering their valuations or social incentives to vaccinate?
• Especially if we can’t say much about the mechanisms, the policy implications are not clear. Moreover, it seems impractical and unsustainable to suggest that governments should maintain low vaccine supplies at all times or that they should lie to people about supplies being low. So, while it’s useful to know that low availability affect vaccine uptake, its seem like an inherently transient factor with few policy implications or aggregate uptake implications.

**I have aimed to address both concerns by re-writing the discussion section of the manuscript:**

**“(…) This has left the impression that the scarcity of SARS-CoV-2 vaccines may increase the willingness of individuals to be vaccinated. However, this observation at the macro level did not allow for any causal conclusions on the micro level of vaccination behavior (Ramström, 2018). For this reason, an experimental study was conducted to test whether a scarcity impression of the SARS-CoV-2 vaccine can indeed lead to an increased willingness to vaccinate.**

**The results of the presented study support the idea that perceived scarcity of SARS-CoV-2 vaccines may be *one* driving factor for increasing people’s vaccination willingness. Of course, only limited conclusions about the complexity of vaccination decisions can be drawn from such a small study, and a cross-sectional experiment like this one only represents a momentary snapshot, through which it is not possible to construct a comprehensive explanation of the processes of the last weeks and months.**

**That said, the results of this study do fit into a larger picture of scarcity effects in varying fields and confirm that scarcity information has a promoting effect on the demand of a good (Aggarwal et al., 2011; De Janvry et al., 2006; Gierl & Huettl, 2010; Jang et al., 2015). Another question is whether such effects are desired in the vaccination context. On the one hand, in the fight against the COVID-19 pandemic, it is highly desirable to achieve herd immunity as quickly as possible by maximizing vaccination rates. On the other hand, scarcity effects represent a rather superficial form of information processing (see, e.g., Mittone & Savadori, 2009; Verhallen, 1982). In this vein, Pereira and colleagues (2021) found that perceived scarcity of SARS-CoV-2 vaccines increased vaccination willingness among people with low compassion for others, but decreased it among those with high compassion for others. Scarcity induced vaccination willingness may disappear the moment vaccines become widely available. From an informed patient perspective, it would therefore be more desirable to increase vaccination willingness through deeper persuasion (see, e.g., Argote et al., 2021; Gowda et al., 2013).”**

Reviewer #5: The article describes a short online experiment to determine if perceived scarcity of COVID-19 vaccines has an impact on vaccination willingness in Germany, during May 2021. The experiment was pre-registered, and provides access to the final data set, the pre-registered exclusion rules, and the SPSS syntax to redo the calculations, which makes a strong case for the rigourosity of the analysis.

I have minor comments about the manuscript that I'm going to list as follows:

1) You are reporting Wilks Lambda and Eta square, but you are not interpreting nor commenting on those results in your discussion. Perhaps it would be better to add a short interpretation in both cases.

**Thanks for the advice! Both points were addressed by adding further information:**

**„Results showed a small but significant scarcity effect on both dependent variables (with both effect sizes .010 < η^2^ < .039 indicating a small effect, see Lenhard & Lenhard, 2016).“**

**“The overall model test revealed that the treatment differences were also significant on the combination of dependent variables, *F*(2, 171) = 5.91 *p* = .003, η_p_^2^ = .065, Wilk’s** $\boldsymbol{\lambda}$ **= .935, 1-β = .87, indicating a significant explanation of variance on both dependent variables by the experimental treatment.”**

2) Add a comparison between your final sample (N = 175) demographics with German demographics, and add in your discussion a comment on possible biases that the online experiment might have. One of them that is clear to me, it's the participation of more men than women in this study. Are there any references about possible biasses induced by gender?. Also, comment on the limitations and advantages of using a paid website to recruit participants. Are there any references about possible biases on this self recruited population? If so, I think it's important to add them to the manuscript.

**I also added further information on the sample characteristics (l. 198-213):**

**“The exact sample sizes of the excluded sub-groups as well as their characteristics are reported in a CONSORT flow diagram in the online supplement (S1). The final sample size consisted of 175 participants (108 male, 67 female; *M*_age_ = 35.93, *SD* = 9.88; education level: 43.4% university degree, 28% baccalaureate, 20.6% completed apprenticeship; 7.4% other secondary school certificate). The sample was not representative for the entire German population. But, this was not the intention of the study. More importantly, the sample was intended to correspond to the characteristics of the non-vaccinated population at the time of the survey. According to the German Federal Statistical Office, the average age of the sample under research roughly corresponds to that of the unvaccinated population in May 2021 (Statistisches Bundesamt, 2021). There is no available data for the distribution of gender and educational attainment among non-vaccinated individuals in Germany, at the field time of the survey (see Impfdashboard of the Robert-Koch-Institute, 2022). However, later data on vaccination intentions has shown that women tend to be rather unvaccinated than men (Graeber et al., 2021), indicating a potential over-representation of male respondents in the sample under research.“**

**“Prior to the main analysis, I tested whether the experimental conditions varied significantly regarding the distribution of sociodemographic characteristics to control for possible biases. Overall, *n* = 98 respondents were assigned to the scarcity condition and *n* = 77 to the surplus condition. The sub-samples did not significantly differ regarding participants’ age, *M*_age scarcity_ = 36.02, *SD* = 9.80, *M*_age surplus_ = 35.81, *SD* = 10.05, *t*(173) = 0.14, *p* = .887, nor gender, χ^2^ (1, *N* = 175) = 0.23, *p* = .642, nor educational attainment, χ^2^ (6, *N* = 175) = 3.18, *p* = .786. With these results, systematic biases between the treatment conditions can be excluded, which allows potential treatment effects to be attributed to the experimental manipulation. In addition, results of a *t*-test between the scarcity and surplus condition regarding the manipulation check variable revealed that individuals in the scarcity condition perceived the supply situation as significantly worse than those in the surplus condition, *M*_scarcity_ = 2.93, *SD* = 1.10, *M*_surplus_ = 4.58, *SD* = 0.78, *t*(172) = -11.14, *p* < .001.“**

3) For Table 1, report exact p-values along with the Eta square for treatment. This is the main result of the analysis, hence saying p<0.05 or p<0.01 may not be enough.

**The exact p-values have been added now (l. 205-220):
“The sub-samples did not significantly differ regarding participants’ age, *M*_age scarcity_ = 36.02, *SD* = 9.80, *M*_age surplus_ = 35.81, *SD* = 10.05, *t*(173) = 0.14, *p* = .887, nor gender, χ^2^ (1, *N* = 175) = 0.23, *p* = .642, nor educational attainment, χ^2^ (6, *N* = 175) = 3.18, *p* = .786. With these results, systematic biases between the treatment conditions can be excluded, which allows potential treatment effects to be attributed to the experimental manipulation. In addition, results of a *t*-test between the scarcity and surplus condition regarding the manipulation check variable revealed that individuals in the scarcity condition perceived the supply situation as significantly worse than those in the surplus condition, *M*_scarcity_ = 2.93, *SD* = 1.10, *M*_surplus_ = 4.58, *SD* = 0.78, *t*(172) = -11.14, *p* < .001.**

**To compare both experimental treatment conditions, a one-way MANOVA was conducted. Results showed a small but significant scarcity effect on both dependent variables (with both effect sizes .010 < η^2^ < .039 indicating a small effect, see Lenhard & Lenhard, 2016). In line with the *Main Hypothesis*, participants in the scarcity condition reported significantly higher willingness to get vaccinated against the SARS-CoV-2 virus compared to those in the surplus condition, *M*_scarcity_ = 4.91, *SD* = 1.88, *M*_surplus_ = 4.31, *SD* = 2.07, *F*(1,174) = 4.03, *p* = .043, η^2^ = .023 (see figure 1).“**

4) A descriptive plot for the Vaccination willingness and Anger measures according to each of the covariates could be useful to describe the data set before entering into the analysis.

This has been also added (l. 139-152):

***“Dependent variables*. Participants’ vaccination willingness was measured by the mean value of the following three items: “Will you get vaccinated as soon as possible?”; “I will be trying harder to get a vaccination appointment in the near future.”; “If necessary, I will use various channels (e.g., primary physician and vaccination centers) to get a vaccination appointment.”; reaching from 1 = “very unlikely” to 7 = “very likely”, Cronbach’s α = .93, *M* = 4.65, *SD* = 1.98. The naming of the dependent variables differs from the preregistration. In the preregistration, vaccination willingness and appointment intention were mentioned separately. For reasons of conceptual overlap and high internal consistency, the items were combined into one general vaccination willingness scale.**

**For the explorative analysis, participants were also asked to answer whether they are angered by the debate over relaxations for vaccinated individuals (“I am angry about the current debate over advantages and liberties for vaccinated people,” 1 = “completely disagree” to 7 = “completely agree”, *M* = 4.44, *SD* = 2.12). This item was added for explorative reasons, but was not part of the preregistration.”**

Reviewer #6**:** Summary

The study explores the association between the perceived scarcity of the COVID-19 vaccine and people’s willingness to get vaccinated. The topic is very timely and is of significant importance. However, there are a number of issues that need to clarified about the study design to ensure its validity and about its limitations and the effect of these limitations on the results and their interpretation. To account for potential confounders and quantify the effect size accurately, I strongly recommend Author perform multivariate regression analysis, instead of one-way MANOVA. If this cannot be done, Author should explain the reasons in the discussion as it seems to be a better study design to examine the association between perceived scarcity of vaccines and willingness to get vaccinated. To be acceptable for publication, the manuscript requires substantial revision as follows.

Introduction

Author described in detail the impact of vaccine scarcity on consumer demand and its applicability to the current context of COVID-19 vaccine roll-out. In general, the introduction described the current situation in Germany well to provide context to the study. However, all these factors mentioned in the introduction should be accounted for in the analysis. Also, there is little justification for the reason why Author decided to measure the “anger” as an exploratory analysis (i.e., why is it of importance and what is its relevant to the main hypothesis?).

Page 3, paragraph starting with “As for the context of vaccines, …”: This sentence does not appear to have the correct citation. Pls check and include an appropriate citation.

**The citation was added in l. 48.**

Page 4, sentence starting with “At first, those countries…” requires citation.

**Ritchie et al., 2020 was added as an appropriate citation in this context (l. 58).**

Page 4, sentence starting with “However, despite all these initial obstacles, …”: When you say “the period of the national vaccine shortage”, please be explicit and state the exact period in the text.

**There is no concrete time range when the shortage began or ended, but based on further publications on this phenomenon, I added the information that this took place in the first half of the year (l. 70-72).**

**„However, despite all these initial obstacles, the overall level of vaccination willingness increased – particularly during the period of the national vaccine shortage in Germany in the first half of the 2021 (ARD-Deutschlandtrend, 2021; Cassel & Ulrich, 2021).“**

Page 5, paragraph starting with “In addition to the test of a scarcity…”: This is the first time Author introduced the debate over relaxation of measures for those vaccinated. If this is one of the outcome measures you aim to test in your study, please provide more context as to its importance before introducing a question related to it.

**As outlined in the manuscript – which is a *short report* – this was an explorative question and has now been embedded in the following information on why this variable was added (l. 87-92):**

**“In Germany, this debate first arose in February 2021, when a sizable portion of the prioritized groups (mainly high age groups) had already been vaccinated. However, the debate became particularly heated and polarized between April and May 2021, when relaxations for vaccinated people were legally adopted despite the fact that a majority of the population had still not yet been vaccinated due to the nationwide shortage. In national opinion polls, many expressed their opposition to these relaxations (see Tagesschau, 2021).”**

Methods

Fundamentally, I am concerned with the validity of the study design.
1) Author should not describe this study as an experimental study. There is no counterfactual (pre-/post-test or treatment vs. control) group or randomization. The study appears to be a cross-sectional study.

**I see that it may have been not clear enough in the procedure section that the study clearly was an experiment as participants were randomly assigned to one of the experimental conditions. This was clarified by the following part (l. 106-111):**

**“Then, participants were randomly assigned to a short text claiming either that their county would be provided with a below average amount of vaccine in the coming weeks or an above average amount (scarcity vs. surplus condition). The county-level manipulation was chosen because people are more likely to be well informed about the vaccine shortage at the national level, but less so about local supplies, as those are less often reported in the media.”**

2) The ‘treatment’ described in the study is not a true treatment. The instant exposure to crafted information on vaccine surplus/scarcity is unlikely to lead to an immediate change in participants’ perception about vaccination. Therefore, exposure to treatment -> associated outcome relationship here is not established.

**Typically, in psychological research experimental conditions are also referred to as “treatments” (see Myers & Hansen, 2012, p. 22). To guarantee that the experimental manipulation of participants’ level of perceived scarcity has worked, a manipulation check was performed after the assessment of the dependent variables. This allows to attribute detected differences on the dependent variables to the previous experimental treatment (Fiedler et al., 2021) – especially when the sample is balanced on further variables, such as socio-demographic control variables, which is the case in the underlying study. This was added by the following section (l. 174-183):**

**“Prior to the main analysis, I tested whether the experimental conditions varied significantly regarding the distribution of sociodemographic characteristics to control for possible biases. Overall, *n* = 98 respondents were assigned to the scarcity condition and *n* = 77 to the surplus condition. The sub-samples did not significantly differ regarding participants’ age, *M*_age scarcity_ = 36.02, *SD* = 9.80, *M*_age surplus_ = 35.81, *SD* = 10.05, *t*(173) = 0.14, *p* = .887, nor gender, χ^2^ (1, *N* = 175) = 0.23, *p* = .642, nor educational attainment, χ^2^ (6, *N* = 175) = 3.18, *p* = .786. With this result, systematic biases between treatment conditions can be excluded, which allows potential treatment effects to be attributed to the experimental manipulation.”**

3) Similarly, the study design does not rule out the possibility that the participants’ willingness to be vaccinated was established prior to their exposure to information on vaccine surplus/scarcity. There is no pre-/post- comparison to establish the temporal relationship between the exposure and the outcome. The current results without this does not support a causal relationship.

**The experimental method, in particular, is the most superior method for enabling causal inference. Unlike correlational data, for example, experiments allow changes in the dependent variable to be attributed to the experimental manipulation. In particular, if it was controlled for that the experimental treatment was successful and if the samples are balanced on additional variables, as is both the case in the present study. I would like to quote Myers and Hansen (2012, p. 22-23) at this point to illustrate what the experimental method means in psychology:**

**“A psychology experiment is a controlled procedure in which at least two different treatment conditions are applied to subjects. The subjects’ behaviors are then measured and compared to test a hypothesis about the effects of those treatments on behavior. Note that we must have at least two different treatments so that we can compare behavior under varied conditions and observe the way behavior changes as the treatment conditions change.”**

**And p. 24:**

**“The greatest value of the psychology experiment is that, within the experiment, we can infer a cause-and-effect relationship between the antecedent conditions and the subjects’ behaviors. If the XYZ set of antecedents always leads to a particular behavior, whereas other treatments do not, we can infer that XYZ *causes* the behavior.”**

**As all these criteria are met by the presented study, this is why the study can be clearly categorized as an experiment.**

4) In addition, Author used one-way MANOVA to test the hypothesis, which does not account for the potential confounding effects. This way, it cannot be ruled out that the participants’ willingness to be vaccinated is driven/mediated by other factors than the perceived scarcity of vaccines. This contradicts what was stated in the introduction and the discussion that vaccine efficacy or side effects also can drive people’s willingness to be vaccinated. Author needs to justify why one-way MANOVA was used on the set of outcome variables. Author should also separately examine and present in a table whether there are any groups differences with regard to demographic and other confounding variables to bolster the findings.
5) Lastly, it is unclear why Author did not choose to perform multivariate regression analysis on each of the outcome variables to account for potential confounding factors. This should be explained in the discussion.

**I will answer to both concerns at once.**

**From a mathematical point of view, a MANOVA and a multivariate regression are basically the same (see Franco, 2019). Both belong to the family of general linear models. The main difference is that the independent variables used in the MANOVA must be categorial, whereas independent variables of regression models are allowed to be metric. When performing an experiment with an adequate number of participants (typically > 30 per condition), a general assumption is that potential moderators (i.e., confounding or influencing variables) will be equally distributed across the experimental conditions. This allows experimentally working researchers to draw causal inference that variations in the outcome variables are mainly due the experimentally varied factor (in this case scarcity versus surplus information). The statistical model of choice in this case is usually (M)ANOVA because the treatment variable is categorical. A regression, on the other hand, is particularly suitable when the influence of a variable is tested in interaction or with concurrent effects of many other variables. However, since no further control variables were collected in this study and the design is not correlative, I consider MANOVA to be indicated.**

Currently, your methods section is a lengthy walk-through of the online survey. Some information you included, such as how participants were “thanked for their participation”, is not essential. Rather than describing the survey procedure in detail, please provide the well-articulated summary of what you were trying to measure, how they were measured in the survey (i.e. type of question or scale used), and how they were coded in the data. Also, whenever a technical term is introduced, spell it out the first time it was used, and explain what it is used for.

**Also in order to meet previous points raised by the other reviewers, the method section was clarified and rephrased (**

Page 5, sentence starting with “Invitation mails were sent out…”: What was the sampling pool for this invitation? what is meant by “weighted by” age and gender?

**I revised this part in order to be more precise (l. 99-111):**

**“Participants were recruited between May 07 and 19, 2021 via a German crowdsourcing platform ([Clickworker,](https://www.clickworker.de) 2021) and were compensated with EUR 0.60 for a maximum duration of 3 minutes. Invitation mails were sent out by the crowdsourcing platform and have been automatically weighted by the age and gender distribution of the German population. At the beginning of the online survey, participants confirmed the informed consent. As the treatment contained mild deception, the informed consent contained information that they might be deceived during participation and that the true purpose of the study could not be explained until the end.“**

Page 6, paragraph starting with “Participants then answered questions…”: What are the dependent variables and what do you mean by ‘manipulation check’? What do you mean when participants were not directed to the “participation code”? What is the “debriefing information”? Are these important facts to be mentioned in this section?

**I agree that this might have been not clear enough, so that I revised the section as follows (l. 129-135):**

**“Participants then answered questions on the dependent variables and the manipulation check, namely how good or bad they perceived the supply situation of their county. At the end of the survey, respondents were thanked for their participation and were informed of the intention of the study. Since deception took place, participants were not directed to the last survey page including the participation code for the payment until they indicated that they had read and understood the debriefing information. This has been implemented for ethical reasons, so that participants would be informed about the true purpose of the study in any case.“**

Page 7, “Dependent variables”:
1) What was your rationale to include these three questions to measure the outcomes? Are these questions from a validated instrument to measure willingness to receive vaccination? (If yes, please cite the work.)

**These items were developed in order to measure vaccination willingness in a specific study context. For this purpose, the items were not adapted from previous research, but self-developed. Vaccination willingness is often measured by a single item. However, there is rising debate of the use of single item measures in psychological research (see Allen et al., 2022 for a discussion). As this context was seen as one where multiple items could have been easily assessed, I therefore decided to use more than one item.**

2) Why is the scale from 1 – 7? It appears to be unconventional.

**Measuring vaccination willingness and other related concepts on a 7-point Likert scale is absolutely common (see Böhm et al., 2019; Huang et al., 2021; Sprengholz et al., 2021).**

3) Did you combine the three questions to form one outcome measure scale? If so, how did you combine them? (i.e., average of 3, sum of 3, etc.).
4) What is the ‘alpha’? (if it is Cronbach’s alpha, you need to specify it explicitly in the text and explain what it means)

**Both points have been addressed by re-writing the section as follows (l. 127-135):**

**“Participants’ vaccination willingness was measured the mean value of the following three items: “Will you get vaccinated as soon as possible?”; “I will be trying harder to get a vaccination appointment in the near future.”; “If necessary, I will use various channels (e.g., primary physician and vaccination centers) to get a vaccination appointment.”; reaching from 1 = “very unlikely” to 7 = “very likely”, Cronbach’s α = .93, *M* = 4.65, *SD* = 1.98. The naming of the dependent variables differs from the preregistration. In the preregistration, vaccination willingness and appointment intention were mentioned separately. For reasons of conceptual overlap and high internal consistency, the items were combined into one scale.”**

Page 7, “Manipulation check”:
What is the purpose of measuring this variable?

**A manipulation check is a variable that measures whether the experimental treatment was successful or not, i.e., by measuring the mindset, feelings, cognitions, attitudes or what else was aimed to be manipulated by the experimental treatment (see Fiedler et al., 2022 for a concise discussion on the usefulness of manipulation checks in psychological research).**

Page 7, “Sample size and data analysis”
1) In the first sentence, please clarify what a “large effect size” is and a “small effect size” is. Please provide appropriate ranges.

**To clarify the range of expected effect sizes, this part was revised as follows (l. 147-151):**

**“An a-priori power analysis using G*Power (version 3.1, Faul et al., 2009) for the detection of a medium (*d* = .50 to .70) to large (*d* = .80 to** $\boldsymbol{\geq}$ **1, Lenhardt & Lenhardt, 2016) scarcity effect in a Multivariate Analysis of Variance (MANOVA) including two dependent variables with *d* = .60, 1-β = .95, α = .05 resulted in a required sample size of 158 participants.”**

2) For the power calculation, please specify d, 1-beta, alpha. Also, please spell-out MANOVA when it appears for the first time in the text.

**This was already specified in the previous version, but I have also revised the part regarding the power analysis as follows (l. 144-151):**

**“Since scarcity effects were found to be of large rather than small effect sizes in previous studies (see e.g., Jang et al., 2015; Verhallen, 1982; Worchel et al., 1975), a small sample size was assumed to be sufficient to identify scarcity effects on vaccination willingness. An a-priori power analysis using G*Power (version 3.1, Faul et al., 2009) for the detection of a medium (*d* = .50 to .70) to large (*d* = .80 to** $\boldsymbol{\geq}$ **1, Lenhardt & Lenhardt, 2016) scarcity effect in a Multivariate Analysis of Variance (MANOVA) including two dependent variables with *d* = .60, 1-β = .95, α = .05 resulted in a required sample size of 158 participants. To allow for potential dropout, data from 238 individuals was collected. A one-way MANOVA with treatment (scarcity vs. surplus) as an independent factor, and vaccination willingness and anger towards the debate over relaxations for vaccinated citizens as dependent variables was preregistered.”**

3) In the last sentence, what do you mean when you say “A one-way MANOVA …. was pre-registered?”
**As outlined in the Methods section of the manuscript, the study has been previously preregistered on aspredicted, in order to meet the criteria of open and transparent science (l. 91-93):**

**“**Research question, design, minimum sample size, exclusion criteria, and analysis plan of the study were preregistered on AsPredicted (see <https://aspredicted.org/blind.php?x=kt8us2)>.”

Page 7, “Exclusion criteria”
1) What is a relative speed index? Please describe.

**This comment was addressed by re-writing the phrase (l. 161-164):**

**“**Following the recommendations of Leiner (2019a), participants with a high relative speed index (RSI > 2), which is a measurement provided by the survey platform used (sosci survey, Leiner, 2019b) and is based on processing time that identifies suspicious data patterns associated with poor quality, and reading times of equal to or less than 2 SD below the sample mean were excluded from data analysis.”

2) Spell out SD when it appears for the first time in the text.

**SD means Standard Deviation and is part of the standard language when reporting scientific findings. According to the APA guidelines such everyday technical terms are not used to be spelled out.**

3) The description of the final sample for the study should be provided in the “Results” section using a table including all the variables used in the analysis. Please provide such a table in this section.

**In psychological research, the sample characteristics are typically reported in the Methods section. See Harris (2008) for a guide how to write up experiments in psychology or just the APA guidelines for reporting psychological research (see summary by Bhandari, 2022).**

Results
1) Currently the results section lacks a descriptive table of the sample (a conventional table).

**Again, this is not part of the Results section. However, an additional test was added before reporting the main analysis, in order to show that the samples of both treatment conditions were balanced with regard to the distribution of socio-demographic variables (l. 186-192):**

“Overall, *n* = 98 respondents were assigned to the scarcity condition and *n* = 77 to the surplus condition. The sub-samples did not significantly differ regarding participants’ age, *M*_age scarcity_ = 36.02, *SD* = 9.80, *M*_age surplus_ = 35.81, *SD* = 10.05, *t*(173) = 0.14, *p* = .887, nor gender, χ^2^ (1, *N* = 175) = 0.23, *p* = .642, nor educational attainment, χ^2^ (6, *N* = 175) = 3.18, *p* = .786. With these results, systematic biases between treatment conditions can be excluded, which allows potential treatment effects to be attributed to the experimental manipulation.”

2) In addition, the way in which the results is reported in the text is hard to follow. A table for the results of the one-way MANOVA analysis for each group should be included. Author listed a list of abbreviated terms with figures without explaining what they mean. There is no effect size reported for the treatment, or no justification why the different tests were used to report the results.

**According to the APA guidelines, tables should be avoided if they are unnecessary. As this is a short report, which reports effects on only two dependent variables, an in-text reference to the statistical findings seems to be more adequate. In addition, I always reported the effect sizes in the previous version of the manuscript (e.g., “***M*_scarcity_ = 4.91, *SD* = 1.88, *M*_surplus_ = 4.31, *SD* = 2.07, *F*(1,174) = 4.03, *p* = .043, η^2^ = .023“). η^2^ is a common effect size when reporting the results of a MANOVA.

Discussion

Overall, your discussion is based on the findings from your study to draw general inference on the German population. I am concerned on the validity of the discussion in general because 1) the generalizability of the findings based on a small sample of 175 participants is not discussed; and 2) the current study design does not allow any causal inference. Limitations should also expand further to accommodate the concerns raised in the “Methods” section.

**I agree with the general concerns raised reviewer 6 regarding the interpretation and discussion of the effect found. I therefore established a more cautious tone and elaborated the weaknesses of the study and the need for further research in this area.**

**e.g., by changing the following parts in the discussion:**

**“This has left the impression that the scarcity of SARS-CoV-2 vaccines may increase the willingness of individuals to be vaccinated. However, this observation at the macro level did not allow for any causal conclusions on the micro level of vaccination behavior (Ramström, 2018). For this reason, an experimental study was conducted to test whether a scarcity impression of the SARS-CoV-2 vaccine can indeed lead to an increased willingness to vaccinate.“**

**“The results of the presented study support the idea that perceived scarcity of SARS-CoV-2 vaccines may be *one* driving factor for increasing people’s vaccination willingness. Of course, only limited conclusions about the complexity of vaccination decisions can be drawn from such a small study, and a cross-sectional experiment like this one only represents a momentary snapshot, through which it is not possible to construct a comprehensive explanation of the processes of the last weeks and months.”**

Page 8, sentence starting with “The results support the hypothesis…”: Based on the current study design, there is very little evidence generated from the study to support this statement.

**Based on the fact that the design and the main hypothesis of the paper were preregistered, I would not agree with the statement that there is little evidence based on the design. Especially through the experimental method underlying this study enables a strong test of the scarcity hypothesis.**

Page 9, sentence starting with “Surprisingly, this has not..”: You cannot say this without testing for this. Your current study does not explore the association between the mentioned factors and your dependent variable.

**I agree, this sentence was removed.**

Page 9, sentence starting with “In fact, when some cities…”: This is irrelevant to the findings of this study and should rather presented in the introduction.
Page 9, sentence starting with “On the other hand, scarcity mentalities..”: This sentence is vague and does not add any value to the discussion as it is formed. Author should clarify it.
Page 9, sentence starting with ”Such anger may also quickly…”: Is there any literature documenting such an association? If yes, Author should cite it. This could be an interesting mechanism and Author should clearly state this in the introduction to justify the exploratory question (focusing on measurement of anger).

**Thanks for these comments! I have revised the first part and second part criticized by the reviewer. Also I added a source for the claim I made on reactance.**

**„Such anger may also quickly turn into reactance and a reduced willingness to follow COVID-19 rules (Hajek & Häfner, 2021). This is also in line with findings of a later study by Sprangholz and colleagues (2022) showing that reactance towards COVID-19 measures was higher among people with a high willingness to vaccinate when they were told that vaccines would be scarce in the future.“**

Minor revisions:

No lines numbers provided in the submission file. This made the review of this manuscript difficult which will also make its revision similarly difficult.
Referencing style does not comply with PLOS ONE’s submission guideline, and in-text citation format is inconsistent throughout (i.e., avoid adding hyperlink to a webpage directly in the text, use a citation software so that in-text citations are numbered in Vancouver style).
Footnotes are not permitted as per PLOS One’s guidelines.

**Thanks for these helpful advices! I submitted the manuscript indicating that the correct style will be adapted at a later stage of the manuscript, as is done now.**

**I added lines to the manuscript and changed the citation style.**

Overall, writing needs to be improved in terms of grammar and style. For example, inconsistent use of tense throughout the text; inconsistent use of terminology on “COVID-19 vaccine” (vs. SARS-CoV-2 vaccine)

**Again, thanks for the advice. I have now consistently used the term “COVID-19” when referring to the COVID-19 pandemic and “SARS-CoV-2” to refer to the SARS-CoV-2 vaccine, as this is an adequate notation used by other authors in the field (e.g., Graeber et al., 2021). Also, the manuscript has gone through a native speaker’s (linguist’s) proof reading.**

**References**

Allen, M. S., Iliescu, D., & Greiff, S. (2022). Single Item Measures in Psychological Science. *European Journal of Psychological Assessment, 38*, 1-5. <https://doi.org/10.1027/1015-5759/a000699>

Bhandari, Pritha (February 17, 2022). How to write an APA methods section with examples. <https://www.scribbr.com/apa-style/methods-section/>

Blanca Mena, M. J., Alarcón Postigo, R., Arnau Gras, J., Bono Cabré, R., & Bendayan, R. (2017). Non-normal data: Is ANOVA still a valid option?. *Psicothema*. *29*(4). 552-557. Https://doi.org/10.7334/psicothema2016.383

Böhm, R., Meier, N. W., Groß, M., Korn, L., & Betsch, C. (2019). The willingness to vaccinate increases when vaccination protects others who have low responsibility for not being vaccinated. *Journal of Behavioral Medicine*, *42*(3), 381-391. <https://doi.org/10.1007/s10865-018-9985-9>

Cortina, J. M. (1993). What is coefficient alpha? An examination of theory and applications. Journal of Applied Psychology, 78(1), 98–104. [https://doi.org/10.1037/0021-9010.78.1.98](https://psycnet.apa.org/doi/10.1037/0021-9010.78.1.98)

Draisma, S. & Dijkstra, W. (2004). Response latency and (para)linguistic expressions as indicators of response error. In S. Presser, M. P. Couper, J. T. Lessler, E. Mar- tin, J. Martin, J. M. Rothgeb, & E. Singer (Eds.), *Wiley Series in Survey Methodology. Methods for testing and evaluating survey questionnaires*. Hoboken, NJ: John Wiley & Sons.

Franco, G. (March 22, 2019). What is the difference between ANOVA and regression (and which one to choose). URL: <https://www.statsimprove.com/en/what-is-the-difference-between-anova-and-regression-and-which-one-to-choose/>

Graeber, D., Schmidt-Petri, C., & Schröder, C. (2021). Attitudes on voluntary and mandatory vaccination against COVID-19: Evidence from Germany. PloS one, 16(5), e0248372.

Huang, P. C., Hung, C. H., Kuo, Y. J., Chen, Y. P., Ahorsu, D. K., Yen, C. F., ... & Pakpour, A. H. (2021). Expanding protection motivation theory to explain willingness of COVID-19 vaccination uptake among Taiwanese university students. *Vaccines*, *9*(9), 1046. <https://doi.org/10.3390/vaccines9091046>

Kidd, R. F. (1976). Manipulation checks: Advantage or disadvantage? *Representative Research in Social Psychology, 7*(2), 160–165.

Fiedler, K., McCaughey, L., & Prager, J. (2021). Quo vadis, methodology? The key role of manipulation checks for validity control and quality of science. *Perspectives on Psychological Science*, *16*(4), 816-826. [https://doi.org/10.1177/1745691620970602](https://doi.org/10.1177%2F1745691620970602)

Myers, A., & Hansen, C. H. (2012). Experimental psychology (7th edition). Belmont, CA: Wadsworth.

Nyhus Hagum, C., Tønnessen, E., & AI Shalfawi, S. (2022). Progression in training volume and perceived psychological and physiological training distress in Norwegian student athletes: A cross-sectional study. *PloS one*, *17*(2), e0263575.

Roznik, E. A., & Alford, R. A. (2015). Seasonal ecology and behavior of an endangered rainforest frog (Litoria rheocola) threatened by disease. *PLoS One*, *10*(5), e0127851.

Sprengholz, P., Betsch, C., & Böhm, R. (2021). Reactance revisited: Consequences of mandatory and scarce vaccination in the case of COVID‐19. *Applied Psychology: Health and Well‐Being*, *13*(4), 986-995. <https://doi.org/10.1111/aphw.12285>

Tricarico, E., Borrelli, L., Gherardi, F., & Fiorito, G. (2011). I know my neighbour: individual recognition in Octopus vulgaris. *PloS one*, *6*(4), e18710.

Warren, G. W., & Lofstedt, R. (2021). COVID-19 vaccine rollout risk communication strategies in Europe: a rapid response. *Journal of Risk Research*, *24*(3-4), 369-379. https://doi.org/10.1080/13669877.2020.1870533
