# Supplementary material for: Does perceived scarcity of COVID-19 vaccines increase vaccination willingness? Results of an experimental study with German respondents in times of a national vaccine shortage
Source: PLoS One. 2022 Sep 7;17(9):e0273441. doi: 10.1371/journal.pone.0273441 (PMC9451090; doi:10.1371/journal.pone.0273441)
Supplement: Attachment — Submitted filename: PONE-D-21-30612_review_round2.docx [file pone.0273441.s007.docx]

The author partially, but not sufficiently, addressed the raised points. The manuscript reads much better now with clear description of the methodology and better presentation of the results. I understand the author’s choice of MANOVA given that sociodemographic characteristics were not significantly different across the two groups, therefore were assumed to be relatively well randomized. However, some of the points raised originally on the manuscript still remain unaddressed. At this point, I would not see the current manuscript fit for publication in PLOS ONE for its audience. I would recommend authors to seek for publication opportunities in psychology-centric journals.

Author consistently rebutted the concerns on the description of the methodology referring to psychology literatures/textbooks. However, given the main audience of PLOS ONE is interdisciplinary and the topic is targeting at public health professionals, I still see the choice of “experimental study” and “treatment” inappropriate. Specifically, the author did not respond to the raised concern on whether the instant exposure to the crafted information on vaccine surplus/scarcity can be considered an appropriate exposure to the treatment given that the immediate (and prolonged) change in participants’ perception about vaccination given this exposure is unlikely.

Also, the fact that the study protocol, or its simple summary, was registered to the website the author indicated does not provide any justification on the validity of the hypothesis or the strength of the results. In fact, if this is a true experimental study involving a treatment/intervention on the human subjects, this should have been registered to a proper trial registry (<https://www.who.int/clinical-trials-registry-platform/network/trial-registration>)

The author also claimed the superiority of the experimental study design, however, the author missed the important reason why it is considered superior to the cross-sectional correlational data (i.e. temporality). The pre-study comparison of sociodemographic characteristics is helpful, but is not sufficient to establish a difference between the two groups in the perception towards COVID-19 vaccine is established post treatment / exposure. To ensure the quantification of a causal impact, ensuring the temporality, pre-post comparison is recommended on the same outcome variable.

I still strongly recommend the author to include a table on descriptive statistics. For any observational study (following the STROBE guideline) or randomized controlled trial study (following CONSORT guidelines), the inclusion of descriptive statistics is strongly recommend/required. Author kept insisting it is a “short report”, however there is no submission category for short reports of such kind. If this is to be considered under the research article category, minimum reporting requirements should be met.
