# Supplementary material for: Does perceived scarcity of COVID-19 vaccines increase vaccination willingness? Results of an experimental study with German respondents in times of a national vaccine shortage
Source: PLoS One. 2022 Sep 7;17(9):e0273441. doi: 10.1371/journal.pone.0273441 (PMC9451090; doi:10.1371/journal.pone.0273441)
Supplement: Attachment — Submitted filename: 2022 Response_Letter_2_PONE.docx [file pone.0273441.s008.docx]

Dear Prof. Abd El-Aty,

I would like to resubmit the revised version of the manuscript entitled “Does perceived scarcity of COVID-19 vaccines increase vaccination willingness? Results of an experimental study with German respondents in times of a national vaccine shortage.” (PONE-D-21-30612)

I would like to thank you and the reviewers for your assessment and evaluation of the manuscript!

First of all, I am happy to see that 4 of the 6 reviewers agree with the revised version of the manuscript and have accepted the paper for publication in PloS One!

Regarding the remaining 2 reviews, I have made some additional major revisions which I shortly want to outline here. First, I have added results from additional analyses in which I used a weighting variable to overcome the gender bias of the sample. The results remained the same when using this weighting procedure, so I reported them in the supplementary file. In addition, I added some more arguments in the manuscript to justify why I used the manipulation check as an exclusion criterion. I also argue within this response letter why using this preregistered exclusion criterion is a necessary component in this context of experimental research. I also argue, why I think that having summarized the vaccination willingness items to one scale was useful.

Again, I want to highlight that this is a short report on the results of a small preregistered study on scarcity effects in the context of the COVID-19 pandemic. As such one, to my experience and opinion, it has several strengths which I am not used to typically see in this category of research articles. First, the study has been preregistered to the data collection and the results support the preregistered hypothesis. Second, the preregistration, data, and syntax have been immediately made public after data collection in order to meet the open science criteria. Third, the short report deals with a (still) highly topical issue, as next waves of vaccination shortage can be expected when the omicron-specific SARS-CoV-2 vaccines will be available.

Attached is a revised version of the manuscript with tracked changes as well as an untracked version.

In this response letter, I have bolded my responses to the reviewers’ comments.

Thank you for giving me the chance to having revised and resubmitted the manuscript!

Best,

The author

**Reviewer #4:** The author does a reasonable job in addressing many of the issues I raised, especially regarding providing additional information about the experiment. If other reviewers and the editors are ok with a paper that has little to say about mechanisms and external validity, I do not want to stand in the way of its publication. However, there are two issues with the empirical analysis that I have major concerns about, and cannot support the publication of a paper on a topic with important public health implications in a wide-read scientific journal until they are addressed. At the moment, the results are not credible enough in my view. Fortunately, there are simple fixes that I propose below.

First, I remain seriously concerned that the exclusion of people that the manipulation did not work for is introducing bias by conditioning the sample on a function of treatment. Specifically, the article notes that: “participants for whom the scarcity vs. surplus manipulation did not work, i.e., individuals in the scarcity condition who perceived the availability to be high (perceived vaccine availability > 4) and individuals in the surplus condition who perceived the availability to be low (perceived vaccine availability < 4), were excluded from the data analysis.” Although the preregistration stated “participants for whom the treatment failed”, it did not prespecify how this would be done.

**I want to answer to this concern by drawing a broader picture on the nature of experimental research in psychology/behavioral economics/communication science. The basic idea of an experiment is that a potential causal relationship between two variables x (explanans) and y (explanandum) is tested by varying different levels of x, and randomly assigning them to the participants. Afterwards, it is tested whether these different levels of x resulted in different degrees of y. In the case of the underlying study x is scarcity versus surplus information and y is vaccination willingness and anger about relaxations. Different from medical research, for instance, in psychological/behavioral economics/communication science research, experimental conditions typically aim to (temporally) change or affect participants’ perceptions of a situation, their cognitive states, as well as feelings, thoughts or mindsets about something. However, the success of the experimental manipulation, which often is done by just exposing participants to a short newspaper expert, is conditioned to so many external variables, such as whether participants draw attention to the presented piece of information, whether they have fully read the information (processing time), whether they have understood it correctly, and – very importantly – whether they believe in the information or not. As the presented research dealt with a highly topical and widely discussed issue, and thereby aimed to increase the ecological validity and applicability of the study, a test of whether the experimental manipulation worked among participants or not was more than necessary. In the preregistration form this was indicated by defining the exclusion criterion of excluding those for whom the manipulation did not work. I think from a pure logical perspective, this means the experimental manipulation worked for participants in the scarcity condition who perceived the availability of COVID-19 vaccines to be bad *and* participants in the surplus condition who perceived the availability to be good. In contrast, the manipulation failed for participants in the scarcity condition who perceived the availability of COVID-19 vaccines to be good *and* participants in the surplus condition who perceived the availability to be bad. Empirically, as a logical consequence, if the manipulation check variable of perceived vaccine availability reaches from 1 (very bad) to 7 (very good), a failed manipulation is operationalized by:**

**Participants in the scarcity condition who perceive the availability to be good, i.e., higher than 4 (scale mean).**

**Participants in the surplus condition who perceive the availability to be bad, i.e., lower than 4 (scale mean).**

This is simply bad practice, and seriously risks generating biased estimates. Let me explain. If the baseline mean is below 4 (which it seems to be), there is a risk that the experimental design is broken because participants in the scarcity treatment is less likely to be dropped because they are more likely to have a score below 4 than participants surplus treatment are likely to have a score above 4. Indeed, this fits with the fairly substantial imbalance across scarcity (N=98) and surplus conditions (N=77) after this exclusion criteria is applied, despite even probabilities of treatment assignment (as I understand it). I imagine that this 44% to 56% split would be statistically distinguishable from 50:50 in even this small sample. The consequence of this is that the randomization is likely to be undone because people in the treatment and control groups possess different baseline beliefs due to the exclusion criterion; this may in turn drive differences in observed outcomes.

**There is a lot of high impact literature claiming that manipulation checks in experimental research are more than ever needed, especially behind the background of the replication crisis in psychological and behavioral science (e.g., Fiedler et al., 2021; Gollwitzer & Schwabe, 2021). The experimental design is not „broken" by this practice, but is guaranteed by the manipulation check in the first place. Making use of the manipulation check as an exclusion criterion (which was preregistered) does not confound the results, but in the opposite, it saves the study from being confounded by other background variables, such as high knowledge about the actual supply situation or string trust in the responsible local authorities to deal with the situation. Only by implementing the manipulation check as an exclusion criterion it is guaranteed that observed changes in the dependent variable can be attributed to previous variations of the independent variable. Or to say it with the words of Fiedler et al. (2021, p. 818):**

**“MCs are critical for the viability of the logical premise of a theoretical hypothesis H: Δx → Δy, which predicts a shift in the dependent variable (DV) y (Δy) given a shift in independent variable (IV) x (Δx). This prediction is logically contingent on the premise that an experimental treatment actually succeeds in inducing the intended Δx shift. Without that premise, predicting an effect Δy is unwarranted.”**

**In a similar vein, Gollwitzer and Schwabe (2021) argue that manipulation checks are particularly recommended for the use of subtle manipulations (p. 6).**

**I don’t share R4’s assessment in this aspect. However, I outlined in more detail and with a stronger reference to existing research, why I used the manipulation check as an exclusion criterion and how this can help to increase data quality (p. 7).**

**“There is a growing body of criticism on doing experimental research without controlling for a successful experimental manipulation within the sample under research [32-33]. Given that people’s prior knowledge and information could have strongly influenced whether people believed the treatment texts or not, we preregistered the manipulation check variable as an exclusion criterion, as done in other experimental research [34-36].”**

**Fiedler, K., McCaughey, L., & Prager, J. (2021). Quo vadis, methodology? The key role of manipulation checks for validity control and quality of science. *Perspectives on Psychological Science*, *16*(4), 816-826.** [**https://doi.org/10.1177/1745691620970602**](https://doi.org/10.1177/1745691620970602)

**Gollwitzer, M., & Schwabe, J. (2020). Context dependency as a Predictor of Replicability. *Review of General Psychology*, 10892680211015635.** [**https://doi.org/10.1177/10892680211015635**](https://doi.org/10.1177/10892680211015635)

The paper now conducts balance tests over age, gender, and education, and finds no difference; while this is somewhat comforting, these variables are not necessarily correlated with the outcomes and inspection of the tables in the supplementary materials do suggest quite large differences in education in magnitude.

**In contrast to the above-mentioned criticism on the use of a manipulation check as an exclusion variable, testing the sample for being balanced in sociodemographic variables was very useful and a good recommendation, as especially age and gender are strong predictors of individuals’ vaccination willingness (Coscia, 2021). With regard to the distribution of the sociodemographics across the experimental conditions, I also wrote in the manuscript (p. 8) that the cell differences in educational attainment between experimental conditions were not statistically significant.**

**“The sub-samples did not significantly differ regarding participants’ age, *M*_age scarcity_ = 36.02, *SD* = 9.80, *M*_age surplus_ = 35.81, *SD* = 10.05, *t*(173) = 0.14, *p* = .887, nor gender, χ^2^ (1, *N* = 175) = 0.23, *p* = .642, nor educational attainment, χ^2^ (6, *N* = 175) = 3.18, *p* = .786.”**

**Nevertheless, regarding the overall bias in gender, i.e., having more male participants in the sample than females, which is in contrast to the gender distribution of the unvaccinated population at the field time of the study, during which according to Coscia (2021) more women were hesitant to vaccinate against COVID-19 than men, I reanalyzed the data by using a weighting variable to correct the oversampling of male non-vaccinated compared to females. According to the data reported by Coscia for Germany, I used the following weighting procedure:**

**As, according to Coscia (2021), 53.11 % of vaccine sceptics were female and 46.89 % were male in the broad time range of the study. The actual gender distribution in the sample was 38.3 % female and 61.7 % male.**

**The weighting variable was calculated following the classical weighting process recommended by IBM SPSS by dividing the target distribution through the actual distribution, resulting in:**

**Weighting factor for female participants: 53.11 / 38.3 = 1.39**

**Weighting factor for male participants: 46.89 / 61.7 = 0.76**

**The results remained the same when using this weighting variable, which was now also mentioned in the manuscript (p. 9).**

**“(…) and when the sample was weighted for participants gender, using a weighting variable which represents the actual gender distribution among vaccination sceptics at the field time of the survey (supplement S4).”**

**Coscia, Verena (October 6, 2021). *Vaccination willingness in Europe: Who are the unvaccinated?* Max Planck Institute for Social Law and Social Policy. https://www.mpisoc.mpg.de/en/newsroom/news/detail/announce/vaccination-willingness-in-europe-who-are-the-unvaccinated/**

Together, these concerns create significant worries about the validity of the results – especially for journal like PLoS One and on an important public health issue.

**In PLoS One more than 45,600 hits appear when searching for articles reporting manipulation checks in their materials and methods section. More than 8,000 hits appear in PloS One when “manipulation check” is paired with “exclusion criteria”. Many of these articles reported that the manipulation check (using different paradigms) was used as an exclusion criterion, e.g.,**

**“An additional 32 participants who failed the memory manipulation check (more than 1 incorrect choice)”**

**Sanchez, C., Sundermeier, B., Gray, K., & Calin-Jageman, R. J. (2017). Direct replication of Gervais & Norenzayan (2012): No evidence that analytic thinking decreases religious belief. *PloS One*, *12*(2), e0172636.**

**“Nineteen patients failed the manipulation check (that is responded to the item “I was ignored” with 1 or 2), and were therefore excluded from the following analyses. The final sample hence comprised 97 patients.”**

**De Rubeis, J., Sütterlin, S., Lange, D., Pawelzik, M., van Randenborgh, A., Victor, D., & Vögele, C. (2016). Attachment status affects heart rate responses to experimental ostracism in inpatients with depression. *PloS One*, *11*(3), e0150375.**

**“Accordingly, complete data from 43 participants were available for analysis (although only 41 were included in the final analysis, due to 2 participants failing the manipulation check—see *Self Report Measures*).”**

**Almarzouki, A. F., Brown, C. A., Brown, R. J., Leung, M. H., & Jones, A. K. (2017). Negative expectations interfere with the analgesic effect of safety cues on pain perception by priming the cortical representation of pain in the midcingulate cortex. *Plos One*, *12*(6), e0180006.**

### “Not all participants from the sampling procedure were included in the experiment. Since the study was conducted online using a self-administered survey experiment with video messages, we administered the manipulations carefully to ensure exposure. One hundred twenty-five respondents, homogenously distributed across conditions, were excluded for one of three reasons: not being able to hear the audio check, reporting having been exposed to the video in full outside of the study, or not being able to play the experiment video: (1) 85 reported they could not hear, and others entered the wrong number during an audio check at the beginning of the study. The 85 participants who could not hear were asked if they had another audio-working device. Of those 85, the 64 who said they did not were dropped from the experiment; others joined with a new device. (2) After viewing the video, 32 respondents stated that they had seen it fully somewhere and were thus dropped from analysis because prior exposure would confound our experimental manipulation. However, 80 respondents who said they remembered seeing part of the video were retained. (3) 22 participants indicated they could not view the entire video and were dropped from analysis due to inadequate exposure to the experimental manipulation. (4) 7 participants who indicated they could not hear the experiment video were dropped as well due to inadequate exposure to the experimental manipulation. Additionally, those who left the audio check question blank (N = 56) at the very beginning of the survey were excluded.

**Due to a survey progression (display logic) error, some participants were able to answer questions without having been shown the experimental module; this reduction in sample size was also random across conditions. Hence, the overall experiment sample size was 2,345. A small number of participants with missing data in dependent and independent variables were also dropped. Final Ns are reported along with the results in** [**Table 1**](https://journals.plos.org/plosone/article?id=10.1371/journal.pone.0248328#pone-0248328-t001)**.”**

Kuru, O., Stecula, D., Lu, H., Ophir, Y., Chan, M. P. S., Winneg, K., ... & Albarracín, D. (2021). The effects of scientific messages and narratives about vaccination. *PloS One*, *16*(3), e0248328.

**I think these examples speak for themselves and rather show that manipulation checks are often used to improve data quality and are by no means used otherwise.**

Fortunately, there is a simple fix here: not imposing this exclusion criteria (although the others are fine). This will immediately restore the experimental properties of the analysis. If the author is concerned about observing small effects because some participants did not internalize the treatment, they should conduct an instrumental variables analysis in addition to reporting the reduced form comparison between treatment and control. Given the simplicity of the solution and obvious risk of bias, I cannot support publication of this article until this more sensible analysis is used for the main results. This is not simply a robustness check, but the proper experimental specification should be the main result.

**I completely see it the other way around. As outlined above, analyzing the data without guaranteeing that the experimental manipulation has worked for the subjects under study would be – as demonstrated above – not reasonable. It is more than clear that the effect diminishes when participants are included who were exposed to scarcity information but did not believe it neither for participants who were in the surplus condition and did not believe in this information. This is simply a cutoff of research with high ecological validity which is situated in a current context. There one must simply exclude participants who do not believe in the presented information. Otherwise, the sub-samples are highly confounded. This is not a disadvantage of the conducted research, but a circumstance to which applied research is inevitably exposed.**

Second, and less important, the key dependent variables deviate from the preregistered approach in two respects: (i) there is no mention of creating a mean scale; (ii) one outcome (pursuing various channels) included in the scale in the paper is not mentioned at all in the preregistered analysis. (This is not the exploratory outcome.) This obviously creates concerns regarding researcher degrees of freedom.

**I am sorry, the preregistration file just misses an “e.g.,” when I referred to vaccination willingness as it is one line below when I mentioned appointment intention. I can understand the reviewer’s criticism, but I think I have mentioned this satisfyingly in the last revision of the manuscript. As there was a high conceptual overlap between appointment intentions and general vaccination willingness, I already outlined why I found it useful to summarize the items. In addition, there is some criticism to use single item measures when multi-item measures are available (e.g., Fisher et al., 2016). I also outlined this in the current revision (see p. 6).**

**“In addition, the items were summarized into one scale as the use of single item measures is increasingly criticized if multi-item measures are possible [28].”**

**Even though I can understand the criticism, I want to say that I cannot do more than being transparent with the deviations from the preregistration – which I am in the whole manuscript.**

**Fisher, G. G., Matthews, R. A., & Gibbons, A. M. (2016). Developing and investigating the use of single-item measures in organizational research. Journal of Occupational Health Psychology, 21(1), 3–23.** [**https://doi.org/10.1037/a0039139**](https://psycnet.apa.org/doi/10.1037/a0039139)

To be more transparent in the presentation of the results, I would like the author to report the effect of manipulation on each of the three outcomes separately (i.e. not just as part of the scale, although it’s ok to keep that). This accords with the method they preregistered. This is important because the outcome that is not mentioned in the preregistration could be driving the results, and seems less conceptually relevant for the study. This is also a simple fix.

**I already did this at a former stage of the review process (see page 9 of the last revised version)!**

Ultimately, I strongly encourage the author to implement these two simple changes that would substantially enhance the credibility of the study’s results. Absent these changes, I would continue to have serious concerns about the validity of the paper’s conclusions.

**I tried to outline above why I disagree with some of the reviewer’s recommendations.**

**Reviewer #6**: The author partially, but not sufficiently, addressed the raised points. The manuscript reads much better now with clear description of the methodology and better presentation of the results. I understand the author’s choice of MANOVA given that sociodemographic characteristics were not significantly different across the two groups, therefore were assumed to be relatively well randomized. However, some of the points raised originally on the manuscript still remain unaddressed. At this point, I would not see the current manuscript fit for publication in PLOS ONE for its audience. I would recommend authors to seek for publication opportunities in psychology-centric journals.

Author consistently rebutted the concerns on the description of the methodology referring to psychology literatures/textbooks. However, given the main audience of PLOS ONE is interdisciplinary and the topic is targeting at public health professionals, I still see the choice of “experimental study” and “treatment” inappropriate.

**Yes, I referred to textbooks and basic literature in the last round of reviews several times, because I obviously had to explain basic knowledge about the rationale of experiments, which was very unfortunate for a peer-review process in an international journal.**

**I'm irritated by R6's comment that this research is not supposed to fit into the PloS One scope. As R6 him/herself mentions, PloS One is an interdisciplinary journal that has a high profile ESPECIALLY in Psychology and Health Communication. For the keywords vaccination willingness alone, more than 25,000 hits can be found at PloS One! Some of the articles from PloS One cited in my manuscript are dedicated to a very similar topic. Therefore, I see R6's comment as unwarranted and unsupported.**

Specifically, the author did not respond to the raised concern on whether the instant exposure to the crafted information on vaccine surplus/scarcity can be considered an appropriate exposure to the treatment given that the immediate (and prolonged) change in participants’ perception about vaccination given this exposure is unlikely.

**I don’t see this comment well established, as I have discussed this point authoritatively in the revised version of the manuscript and in the previous response letter, justifying why the experimental manipulation can be considered warranted and that other research has now used similar manipulations of vaccine scarcity – even in PloS One.**

Also, the fact that the study protocol, or its simple summary, was registered to the website the author indicated does not provide any justification on the validity of the hypothesis or the strength of the results. In fact, if this is a true experimental study involving a treatment/intervention on the human subjects, this should have been registered to a proper trial registry (<https://www.who.int/clinical-trials-registry-platform/network/trial-registration>)

**I clearly have to disagree with the reviewer. More than 71,000 hits appear in PloS One when I search for articles including the keywords “registered the open science framework”! The Open Science Framework is the best-known Open Science Platform in Cognitive and Behavioral Sciences and is a critical part of the Open Science movement (Open Science Collaboration, 2015). This study is not a clinical trial study!!! I checked the link and this recommendation is completely inappropriate as it is related to medical/clinical research – this manuscript refers to opinion measures not clinical measures.**

**Open Science Collaboration. (2015). Estimating the reproducibility of psychological science. *Science*, *349*(6251), aac4716.**

The author also claimed the superiority of the experimental study design, however, the author missed the important reason why it is considered superior to the cross-sectional correlational data (i.e. temporality). The pre-study comparison of sociodemographic characteristics is helpful, but is not sufficient to establish a difference between the two groups in the perception towards COVID-19 vaccine is established post treatment / exposure. To ensure the quantification of a causal impact, ensuring the temporality, pre-post comparison is recommended on the same outcome variable.

**I already explained this in the last round of reviews. Cross-sectional / correlational data must NEVER be interpreted causally! In contrast, experimental research allows for an adequate test of whether an independent variably causally affects a dependent variable. Also, a pre-post design is a repetition of measurement but per definitionem not necessarily an experiment! I have the impression that the reviewer mixes some things up, here.**

I still strongly recommend the author to include a table on descriptive statistics. For any observational study (following the STROBE guideline) or randomized controlled trial study (following CONSORT guidelines), the inclusion of descriptive statistics is strongly recommend/required. Author kept insisting it is a “short report”, however there is no submission category for short reports of such kind. If this is to be considered under the research article category, minimum reporting requirements should be met.

**A table of the descriptive statistic was already part of the revised manuscript (see p. 7 and Supplement 1 of the last revision)!**
