# Supplementary material for: Does perceived scarcity of COVID-19 vaccines increase vaccination willingness? Results of an experimental study with German respondents in times of a national vaccine shortage
Source: PLoS One. 2022 Sep 7;17(9):e0273441. doi: 10.1371/journal.pone.0273441 (PMC9451090; doi:10.1371/journal.pone.0273441)
Supplement: Attachment — Submitted filename: PONE_Response_3.docx [file pone.0273441.s009.docx]

Dear Dr. A. M. Abd El-Aty,

Thank you for your invitation of revising and resubmitting the manuscript titled “Does perceived scarcity of COVID-19 vaccines increase vaccination willingness? Results of an experimental study with German respondents in times of a national vaccine shortage.”

I have revised the last version of the manuscript in accordance to many of the reviewers’ comments.

Regarding some concerns on the methodological appropriateness, I have different opinions as one of the reviewers, on which I want to reply in more detail in this response letter. More precisely, I have undertaken several analyses to make sure that the chosen exclusion criteria have not led to any bias between included and excluded participants. I have reported these additional analyses in the supplements of the manuscripts, following the CONSORT flow-chart.

In the following, I want to outline point by point, how I addressed the reviewers’ concerns. Therefore, I printed my answers in bold:

Reviewer #2: - the author should include lists of abbreviations
-the author should lists source of data, method of data collection, statistical model and statistical software used for analysis

**A list of abbreviations is now attached in the notes section of the manuscript.**

Reviewer #4: Thank you for the detailed response to my comments, and those of reviewer 6. I think the author has made useful progress in this revision. However, the important point about introducing bias is still not properly addressed. I expand on why below. I also think that the author can still be clearer about what is and is not registered in the main paper, but this is a more minor concern.

ISSUE 1: INTRODUCING BIAS BY POST-TREATMENT SAMPLE SELECTION

I remain unsatisfied with the author’s response on my most important point regarding the individuals that were dropped from the sample. While I appreciate the author’s extended response and I appreciate that there are differences across disciplines, everything in the modern causal inference toolkit in econometrics and program evaluation says that restricting the sample on the basis of post-treatment outcome is not good practice and can introduce bias.

**Sorry, but I have to disagree. This is not the one and only current opinion in econometrics and program evaluation which the reviewer is describing here. As I outlined in my last response, this method is quite often practiced (also in PLOS One) and after my (and other authors’) opinion, the aptness of post-treatment selection widely depends on questions like how is the treatment induced and how can its success be proven/measured? I don’t agree that there is an absolute “the-one-and-only-approach” to handle exclusion criteria. As outlined below, I am able to show that there are no significant differences between the sub-samples regarding their socio-demographic composition, so I would claim that Aronow’s et al. (2019) argumentation of possible biases does not apply to the analyses undertaken in this manuscript.**

Indeed, Aronow, Baron, and Pinson (2019) prove exactly this in the context of dropping subjects based on manipulation tests in a recent article in Political Analysis. Quoting from the introduction of their article: “…we show that the practice of dropping subjects based on a manipulation check should generally be avoided. We provide a number of statistical results establishing that doing so can bias estimates or undermine identification of causal effects. We also show that this practice is equivalent to inducing differential attrition across treatment arms, which may induce bias of unknown sign and magnitude. We do not claim that our statistical formulations are particularly novel—they follow from well-known results about conditioning on post-treatment variables and attrition—but, given the prevalence of this practice, we believe that the relationship between these findings and practice in experimentation is underappreciated.” In the context of this paper, my previous review provided an example of how such a bias could arise in this particular application. It also noted that there is a notable difference in the number of treated and control units that emerges after the sample is trimmed, which suggests the risk of bias.

The author did not seriously engage with this point and seemed to miss my point about manipulation tests. Everyone, including myself, agrees that manipulation tests are useful for validating that the treatment did what it intended to do. There is no disagreement here – with either the author or their quote from Fiedler et al. (2021) or their reference to Gollwitzer and Schwabe (2021) in their response. However, as just explained, dropping observations can bias experimental comparisons when treated and control units are dropped in differential ways, which may occur when they are dropped on the basis of post-treatment outcomes like manipulation checks. I am not saying that there is necessarily bias in this particular case, but there is reason to believe there could be – as explained above and in my previous review.

The author goes on to state that many other papers also drop observations based on the results of manipulation tests. (Note though that many attention tests are not post-treatment or plausibly unrelated to treatment, which makes bias unlikely.) However, I do not believe that others also following bad practice is sufficient reason to risk biasing experimental designs by dropping data on the basis of post-treatment variables – especially when the results could influence policy decisions, and particularly when better methods exist and could easily be implemented instead. Following the seminal econometric textbook by Angrist and Pischke (2008) that builds on Angrist’s Nobel prize-winning work on instrumental variables, I continue to recommend the following:

1. First conduct a reduced form analysis in the *entire* sample that compares treated and control units. The outcomes would be for the manipulation test outcome and the primary outcomes used in the paper. This would yield the average treatment effect. To be clear, this estimate is not confounded; sure, people might not react to treatment for many reasons, but a successful randomization ensures that this is balanced across treatment groups.

**[As I will outline in more detail below, there is no observable confound or bias between the included and excluded individuals.]**

2. If treatment compliance is low, as the author notes, then the natural next step is to use an *instrumental variables* analysis in the full sample. This retains the underlying randomization as the driver of differences in outcomes across treatment groups, but scales up the coefficients to adjust for non-compliance. Under an exclusion restriction (treatment does not affect the outcome except through posterior beliefs) and imposing monotonicity (that treatment conditions cause people to update in a given direction), this also has a causal interpretation – the local average treatment effect among compliers. As far as I understand, this is the author’s target estimand; the sample restriction is designed to estimate effects only for the compliers.
If the results are similar, great – this suggests that the author’s method is not introducing significant bias; then I think it’s fine to report these approaches as robustness checks. If the results differ substantially, there is serious reason to worry about the internal validity of the reported results. In that case, I would favor estimates from the specifications I just proposed, because they do not risk post-treatment bias.

It's also worth noting that the reweighting approach is useful, and helps to make the sample a bit more representative. However, it doesn’t directly address the concern raised - potential imbalances across treatment conditions with respect to observable pre-treatment covariates. Still, as the author notes, there are not significant differences here, even if some differences look somewhat large in magnitude (but perhaps lack of significance might reflect the small sample).
 **I would like to note here that Aronow et al. (2019) argue from a very different perspective. Namely, that it cannot be ruled out that a treatment also influences those participants who failed a manipulation check. In my opinion, this is highly dependent on the variables a researcher examines. For example, if an experimental manipulation aims at mood change, it may be that a manipulation check would exclude a person who is below a certain threshold that the researchers consider to be the minimum necessary mood change. However, the person may have experienced a significant change for him or her intra-individually despite a small change in mood. Their exclusion from the overall sample would thus lead to an underestimation of the global effect and indicate a bias in the sample. Aronow et al. (2019) draw attention to exactly this problem and show, doing a replication of a study without using exclusion criteria, that the effect becomes stronger. In addition, they argue that using manipulation checks as an exclusion criterion can lead to a serious problems and biases when comparing experimental treatment groups including a MC with control conditions not including a MC.**

**In my opinion, however, such generalizations (“Manipulation checks are always inappropriate as inclusion/exclusion criteria.”) do not apply to all types of experimental manipulations. Whether a MC is useful as an exclusion variable depends on the treatment, the measurement of the MC, and the dependent variables. If it is important for the validity of a treatment that the participants process a given text information correctly or believe the information that is presented, then it makes no sense to include persons in the analysis who do not believe the information. If the MC guarantees that participants have read, understood, and believe the content of a treatment text, for example, then that is a legitimate inclusion variable. Why should the treatment have any effect on people who do not believe the treatment text?**

**Of course, this tendency is also evident in my data. If people for whom the MC failed are included in the analyses, then there is no longer a significant treatment effect. But that is perfectly reasonably, because then there are many participants in the sample who may not have read the text, understood it, or simply do not believe the given information. Thus, it makes no sense to report the conduct the analyses over the total sample.**

**In addition, another point made by Aronow et al. (2019) and by Reviewer 4 is that the use of MCs as exclusion criteria can lead to biases between the sub-samples. I checked this statistically and I can say based on statistical comparisons between included and excluded participants that they are not significantly different from each other in socio-demographic variables. In addition, there was no difference between the sub-samples, when checking for interactions between the exclusion criterion and the experimental condition. I also included this note after reporting the CONSORT chart and subsample characteristics in the Supplement:**

**[To test whether there is potential bias through the chosen exclusion criteria, it was tested whether the excluded and included sub-samples significantly differed in their sociodemographic composition. An ANOVA with two factors (experimental condition: scarcity versus surplus; filter: included versus excluded) for testing on age differences between the sub-samples revealed no significant difference between the included and excluded participants, *M_included_* = 35.93, *SD* = 9.88, *M_excluded_* = 35.22, *SD* = 11.33, *F*(1, 138) = 0.26, *p* = 607,** $\boldsymbol{\eta}^{\boldsymbol{2}}$ **= .001. In addition, the interaction between the filter variable and the experimental condition was non-significant, so no age bias was detected between the sub-samples, *F*(1, 138) = 0.09, *p* = 759,** $\boldsymbol{\eta}^{\boldsymbol{2}}$ **< .001. For gender and educational attainment, two Chi-square tests were conducted to test for group differences. Again, there was no difference between the included and excluded participants with regard to gender,** $\boldsymbol{\chi}^{\boldsymbol{2}}$**(*N* = 238) = 0.06, *p* = .803, and educational attainment,** $\boldsymbol{\chi}^{\boldsymbol{2}}$**(*N* = 238) = 3.16, *p* = .788. Also, there was no significant interaction effect between the filter variable and the experimental condition. So, there was no difference between the sub-samples with regard to gender,** ${\boldsymbol{\chi}^{\boldsymbol{2}}}_{\boldsymbol{scarcity}}$**(*N* = 119) = 0.50, *p* = .477,** ${\boldsymbol{\chi}^{\boldsymbol{2}}}_{\boldsymbol{surplus}}$**(*N* = 119) < 0.01, *p* = .982, and educational attainment,** ${\boldsymbol{\chi}^{\boldsymbol{2}}}_{\boldsymbol{scarcity}}$**(*N* = 119) = 1.15, *p* = .949,** ${\boldsymbol{\chi}^{\boldsymbol{2}}}_{\boldsymbol{surplus}}$**(*N* = 238) = 2.43, *p* = .907.]**

**Since this is a criticism where the opinions of reviewer 4 and myself differ widely, I can do nothing else but point out that no relevant bias can be found in my sub-samples and that I have tried to take up reviewer 4’s criticism by focusing more on the weaknesses of the treatment in the discussion and on the fact that more field studies are needed to be able to prove such effects more robustly.**

**[See p. 11-12, l. 253-273: “The results of the presented study support the idea that perceived scarcity of SARS-CoV-2 vaccines may be *one* driving factor for increasing people’s vaccination willingness. Given the simplicity of the design and the rather small size of the effects found, of course, only limited conclusions about the complexity of vaccination decisions can be drawn from such a small study, and a cross-sectional experiment like this one only represents a momentary snapshot, through which it is not possible to construct a comprehensive explanation of the processes of the last weeks and months. Against this background, it also has to be mentioned here that a significant number of participants was excluded as the experimental manipulation has not worked for them, i.e., their perceived availability of COVID-19 vaccines was not affected by the experimental condition. On one hand, using manipulation checks as an exclusion criterion guarantees that the participants under research have been influenced by the given information in the intended direction, which is a necessary precondition to draw any causal conclusions between the experimental variation and the variation on the dependent variables [32-33]. On the other hand, this practice is increasingly criticized in some social science disciplines [43] because of the risk of increasing biases between the sub-samples. As in this study there were no significant differences between the tested sub-samples regarding their sociodemographic composition, the risk of potential bias in this study is comparatively small. Nevertheless, it has to be critically mentioned here that the high number of excluded cases due to a failed manipulation check may speak for the fact that some participants might not have completely read or understood the information, or that they were highly informed about the issue so that the presented information probably has not affected their perception of vaccine availability. This is a clear limitation of the presented study and can only be solved in the future by (a) choosing more distinctive texts to induce scarcity or surplus impressions, (b) conducting quasi-experimental field experiments in which local scarcity situations are covered as independent variables, as well as (c) cross-sectional research on the effects of temporal vaccine scarcity on individuals’ vaccination willingness.”]**

ISSUE 2: TRANSPARENCY ABOUT PREREGISTERED OUTCOMES
With respect to deviations from the pre-analysis plan, I apologize for missing the results in appendix S3. These results do, however, show that the largest effects are on the outcome that was not preregistered, while effects are borderline or null for the two outcomes that were preregistered. Regardless of whether the preregistration plan simply forgot to include this outcome (or an “e.g.”), I think the author should clearly state in the main paper that the index results are driven by an outcome that was not preregistered. I have no problem with publishing finding for outcomes that are not preregistered, but this should be clearly communicated. Readers can make up their mind about whether to downweight the results, but in this case I agree with the author that this outcome is relevant and should be included in the index.

**I addressed these points and made this clear in the manuscript.**

**[p. 7, l. 135-141: “The naming of the dependent variables differs from the preregistration. In the preregistration, vaccination willingness and appointment intention were mentioned separately. For reasons of conceptual overlap and high internal consistency, the items were combined into one general vaccination willingness scale, as the use of single item measures is increasingly criticized if multi-item measures are possible [28]. Also, item three was not explicitly mentioned in the preregistration as the preregistration only included a reference to example items of each scale.”]**
