# Supplementary material for: Does perceived scarcity of COVID-19 vaccines increase vaccination willingness? Results of an experimental study with German respondents in times of a national vaccine shortage
Source: PLoS One. 2022 Sep 7;17(9):e0273441. doi: 10.1371/journal.pone.0273441 (PMC9451090; doi:10.1371/journal.pone.0273441)
Supplement: Attachment — Submitted filename: PONE-D-21-30612R4_ResponseLetter.docx [file pone.0273441.s010.docx]

Dear Dr. A. M. Abd El-Aty,

Thank you for your invitation of revising and resubmitting the manuscript titled “Does perceived scarcity of COVID-19 vaccines increase vaccination willingness? Results of an experimental study with German respondents in times of a national vaccine shortage.”

I was happy to read that Reviewer 2 aims to see the manuscript published (as do now 5 of 6 reviewers)!

With regard to Reviewer 4’s concerns, I keep partly unconvinced. Reviewer 4 would like to see an instrumental variable analyses. I have now gone deeper into the literature on the instrumental variable approach. Below, I answered on Reviewer 4’s concerns point by point and printed my answers in bold.

I added the desired ATE estimation within this response letter. I do not feel that adding the ATE estimation would be something authors typically report in a research paper. So, if I should integrate this estimation in the manuscript, I would prefer to add it to the Supplements.

I shortly want to explain here why I am not convinced that adding any additional instrumental variables analyses to the manuscript would improve its quality.

The instrumental variable approach is typically used in disciplines such as political science or economics in cases when the error term of a regression is correlated with a predictor. Then, a researcher is able to decide to use another related variable (instrumental variable) as a proxy for the actual predictor to avoid the endogeneity problem. Translated to the discussion on the manipulation check variable, I cannot see which instrumental variable I would be able to use in my data to perform such an analysis. For my feeling, an instrumental variables approach would need more related variables which I don’t have in my data.

I have translated the desired instrumental variables analyses in form of an additional mediation model between Treatment 🡪 Perceived vaccine availability (manipulation check variable) 🡪 Vaccination willingness / Anger, which I don’t see to perfectly fit to the method. I don’t feel that this additional analysis improves the manuscript in any way. I rather have the impression that it is still not a good idea to run an analysis across the entire sample. The main reason why I think so is that there are too many possibilities why the manipulation check failed for some participants. The reasons reach from not reading the treatment text to not believing in it or being influenced by exogeneous variable such as structural scarcity. Thus, even a mediation model is likely not to identify a significant indirect effect between the treatment, the perceived vaccine availability and the dependent variables across the entire sample. After having read various articles on the instrumental variable approach, I have the impression that this approach is not indicated for experimental studies like this one. Especially, because I do not see which variable should be used as an IV – if we assume a correlation between the treatment and the error term. Thus, given the underlying data structure, I don’t see that this approach is fully apt for this study. Given my data, I can just say, that the manipulation has worked for some people, but not for others.

So, I still think, that the approach I have chosen and preregistered (!), namely to exclude participants for whom the manipulation did not work, is a more appropriate way to deal with my data. I have controlled for potential biases in previous analyses. Moreover, excluding participants for whom the manipulation did not work is a quite common way in psychological research and (health) communication science. Thus, I don’t feel that this is a justification of rejection – especially, after all extra analyses and revisions I have only undertaken to address Reviewer 4’s exclusive concerns about the manipulation check.

After all the work that I have now put into this manuscript, I would be very grateful if you could make a decision, based on my comments, whether the article can be published soon or not. I think this would be fair for me to know, after now about 1 year of revisions, which were mainly due to address the wishes of 1 out of 6 reviewers.

As I will explain in detail, I do not see that the suggested instrumental variables approach is apt for the experimental method and especially for the study which I presented. I don’t see where this approach meets the characteristics of the method of my study.

I want to thank you in advance for your work on the article and for considering my thoughts on the Reviewer 4’s criticism!

Best wishes,

Julia Schnepf, PhD

Reviewer #2: I have seen the previous manuscript before the authors sent rivised manuscript.all comments are edited. This manuscript should be publish. It is appropriate for publication.

**I thank Reviewer 2 very much for his/her recommendation of publication!**

Reviewer #4: I appreciate the author’s detailed response, and I regard the additional text re the second issue (the outcome that was not preregistered) as resolved. However, I remain concerned that the estimation strategy could be generating biased results.

The author is right that including inattentive respondents would drag down the ATE relative to the ATE for a fully attentive sample, assuming that inattentive respondents experience similar treatment effects as attentive respondents. The author further notes: “If people for whom the MC failed are included in the analyses, then there is no longer a significant treatment effect.” Given this, we cannot tell whether the results go away in the full sample because it includes people that don’t respond to treatment or because the exclusion criteria based on post-treatment manipulation tests introduces bias because, as Aronow et al. (2019) show, this practice can break the randomization.

**As I have previously shown in several comparisons between the sub-samples (excluded versus included etc.), there is no evidence for a break of the randomization in this specific study.**

The natural way to address the author’s point that “there are many participants in the sample who may not have read the text, understood it, or simply do not believe the given information” is to conduct an *instrumental variables* analysis. This will address this issue by estimating the ATE among manipulation check *compliers*. Intuitively, this will rescale the reduced form coefficient to produce what I believe to be the estimand the author care about, but without inducing the risk of bias by differentially dropping treated and control respondents. I have suggested IV approach in all three of my reviews, but the author still has not addressed this suggested solution, which is standard practice for addressing non-compliance.

**I don’t see the beneficial additional information of estimating the average treatment effect (ATE) among participants for whom the MC worked or not. In addition, having read some articles on the estimation of the ATE, I don’t really see how this should help to disentangle the potential reasons of failed manipulation checks in my data or how to increase the information on the effects found in study. However, I performed this analysis.**

**Typically, the ATE estimation is used to compare participants who experienced any form of a treatment (e.g., participants in an experimental condition) or who have a certain characteristic and those who haven’t (e.g., participants in a control group). Mostly, this procedure is used if a researcher assumes an omitted variable to influence the results. For example, if participants with a university degree (Y_1_) and without a university degree (Y_0_) are compared regarding their income level, one omitted variable behind this process could be their general ability, i.e., their choice to do so or not. Thus, the ATE procedure would try to disentangle between criterion (university degree = 1 versus no university degree = 0) and choice (choosing to do a university degree = 1 or not = 0). As data on such omitted variables is mostly missing, ATE estimation is often based on naïve estimators for cells without real data. This is why I struggled to some extent to transfer the ATE estimation to our discussion on the role of the manipulation check.**

**The ATE is defined as the difference between the outcome variable among individuals who received a treatment and those who haven’t.** $\frac{\boldsymbol{1}}{\boldsymbol{N}}\sum_{\boldsymbol{i}} \boldsymbol{(Y}_{\boldsymbol{1}}\left( \boldsymbol{i} \right)\boldsymbol{-}\boldsymbol{Y}_{\boldsymbol{0}}\boldsymbol{(i))}$**. I transferred the idea of the ATE estimation on the underlying phenomenon of the manipulation check as follows. Y_1_(i) = person i’s outcome (vaccination willingness / anger) if he/she is in the scarcity condition; Y_0_(i) = person i’s outcome (vaccination willingness / anger) if he/she is in the surplus condition**

**🡪 Here I see the first problem: In this study, I don’t compare a treatment group with a control group, so I am not convinced that this procedure is fully apt.**

**Nevertheless, if this is defined as Y_1_ and Y_0_, then the ATE = E(Y_1_) – E(Y_0_). E = expected value.**

**Now, we can take the manipulation check into account by defining the ATT and ATN. The ATT is defined as the ATE among the “treated” individuals, in this case participants for whom the manipulation check worked (MC = 1). The ATN is the ATE among the “non-treated” individuals which I translated to participants for whom the manipulation check didn’t work (MC = 0). This results in the 2x2 matrices below.**

**Values of the outcome variable “vaccination willingness” among different subgroups**

|  | **MC = 0** | **MC = 1** |
| --- | --- | --- |
| **Y_0_** | **5.33** | **4.31** |
| **Y_1_** | **5.22** | **4.91** |

**Now, the ATT would be 4.91 – 4.31 = 0.60 and the ATN would result in 5.22 – 5.33 = -0.11.**

**A positive value indicates that scarcity information increased vaccination willingness, a negative value indicates a decrease. If these differences are tested against zero, this results in *t*(155.64) = 1.84, *p* = .076 [p < .10 counts as statistical evidence for one-sided tests] for the ATT and *t*(60) = 0.21, *p* = .836 for the ATN.**

**Values of the outcome variable “anger” among different subgroups**

|  | **MC = 0** | **MC = 1** |
| --- | --- | --- |
| **Y_0_** | **4.56** | **4.05** |
| **Y_1_** | **4.52** | **5.25** |

**Now, the ATT would be 4.75 – 4.05 = 0.70 and the ATN would result in 4.52– 5.56 = -0.04.**

**A positive value indicates that scarcity information increased anger, a negative value indicates a decrease. If these differences are tested against zero, this results in *t*(172) = 2.07, *p* = .040 for the ATT and *t*(60) = 0.37, *p* = .836 for the ATN.**

(Beyond a randomized experiment, an IV analysis imposes two further identifying assumptions: monotonicity and the exclusion restriction. The former is surely satisfied, unless the treatment caused people to believe the opposite of the prompt they received - which would be a bigger problem, but seems unlikely, as the manipulation check suggests. The exclusion restriction requires that the treatment does not affect the outcome except when the manipulation check works. This is a stronger assumption, but would be satisfied if - as the author claims - individuals only did not pass the manipulation because they did not sufficiently engage with the treatment to be affected by it.)

**I think that results outlined above clearly support the idea that the experimental manipulation has affected individuals with a successful MC but not those with a failed MC. So, it can be excluded, that individuals with a failed MC were affected (in any direction) by the treatment.**

In the absence of an IV analysis, which addresses the problem of potentially biased exclusion criteria while adjusting for non-compliance (without dropping observations), it is hard to be confident in the author’s findings. I recognize that there are no imbalances after imposing the exclusion criteria on the three covariates (age, gender, and education) that the author measured. (While comparing the excluded and included samples is an informative diagnostic, the most relevant test here compares the included sample across treatment conditions – i.e. the balance tests that have always been in the manuscript.) However, there could still be imbalances on many other unobservables.

**I see Reviewer 4’s point here. However, the thing is these potential variables were (as he/she correctly states) unobserved. So, I don’t see how I should be able to test any additional differences such as regarding information seeking etc.**

**I have added a lot of possibilities and alternative explanations to the discussion section after the last rounds of revisions.**

There is reason to worry about this because notably more participants drop from the surplus conditions (final sample of 77) than the scarcity condition (final sample of 98). If there was no such differential attrition (instead resulting in an approximate 50:50 split), I would be not be as concerned about the risk of undoing the random assignment.

**I already adjusted for this imbalance by using weighting procedures, and the findings kept stable. When I weighted the cases for a 50:50 distribution, the scarcity effect becomes even more pronounced (see figure below).**

**Results of the MANOVA across the weighted sample**

**In addition, regarding the socio-demographic composition, there is no indicator of a potential bias (which could be, for instance, indicated by differences in educational attainment – which is not the case, as I have added in one of the last revisions). Also, there is no hint that participants for whom the MC failed resulted reported a particularly low vaccination willingness or any “suspicious pattern” indicating that the exclusion criterion could have inherently affected the findings. As visible by the graphics below, vaccination willingness (anger) was even slightly higher in this group.**

If the author is not going to conduct the instrumental variables analysis, I am happy for the editors to adjudicate on this important statistical issue that seems to affect the core findings.

**I have now somehow translated the desired instrumental variables analyses in form of an additional mediation model between Treatment - Perceived vaccine availability (manipulation check variable) - Vaccination willingness / Anger (see Table 1 and 2). However, I do not feel that this additional analysis would improve the manuscript in any way. I rather have the impression that it is still not a good idea to run an analysis across the entire sample.**

**After having read different articles on the instrumental variable approach, there are several assumptions underlying this analysis.**

**1) the analysis is especially recommended when regression models are performed and a predictor is correlated with the error term. 🡪 I don’t see why this should be apt for my analysis. I have performed an experiment, so I experimentally varied the independent variable. Thus, I don’t see why Reviewer 4 assumes an endogeneity problem.**

**2) The instrumental variable should be uncorrelated with the error term. 🡪 If Reviewer 4 thinks that problem 1 is given in my data, I don’t see which instrumental variable should solve this, as I don’t see that there is any instrumental variable that meets the criterion of being fully „exogenous“ in my data set.**

**3) If the IV approach is used to deal with omitted variables, the author should have an idea of which omitted variable may have caused a finding. 🡪 If Reviewer 4 assumes omitted variables, I don’t see how I should be able to identify them within the data I have.**

**Thus, given the underlying data structure and the experimental method, I don’t see that this approach is fully apt for this study. Given my data, I can just say, that the manipulation has worked for some people, but not for others. And that it does only make sense from a causal inference perspective to include those for whom the manipulation has worked.**

**Table 1**

*Results of the mediation model predicting vaccination willingness by treatment via perceived vaccine availability (manipulation check).*

|  | Perceived vaccine availability | | | | |
| --- | --- | --- | --- | --- | --- |
|  | *B* | *SE* | *t* | *p* | 95% *CI* |
| Constant | 3.59 | .09 | 40.39 | <. 001 | 3.41, 3.76 |
| Treatment  (-1 = Surplus, +1 = Scarcity) | -0.22 | .09 | -2.43 | .016 | -0.39, -0.04 |
|  | Vaccination willingness | | | | |
| Constant | 5.32 | .35 | 15.25 | < .001 | 4.63, 6.01 |
| Treatment  (-1 = Surplus, +1 = Scarcity) | 0.12 | .12 | 0.97 | .335 | -0.12, 0.37 |
| Perceived vaccine availability | -0.14 | 0.09 | -1.54 | .125 | -0.32, 0.04 |

*Note*. a-path: *R^2^* = .02, *F*(2,234) = 5.91, *p* = .016; b-path: *R^2^* = .02, *F*(2,233) = 1.93, *p* = .147

Total effect: *c* = 0.15, *SE* = .12, *t* = 1.22, *p* = .224, *95% CI* [-0.09, 0.39]

Indirect effect: *ab* = 0.03, *Boot* *SE* = .02, *Boot CI* [-0.01, 0.01]

*N* = 238

**Table 2**

*Results of the mediation model predicting anger by treatment via perceived vaccine availability (manipulation check).*

|  | Perceived vaccine availability | | | | |
| --- | --- | --- | --- | --- | --- |
|  | *B* | *SE* | *t* | *p* | 95% *CI* |
| Constant | 3.59 | .09 | 40.39 | <. 001 | 3.41, 3.76 |
| Treatment  (-1 = Surplus, +1 = Scarcity) | -0.22 | .09 | -2.43 | .016 | -0.39, -0.04 |
|  | Anger | | | | |
| Constant | 5.33 | .38 | 14.11 | < .001 | 4.59, 6.08 |
| Treatment  (-1 = Surplus, +1 = Scarcity) | 0.19 | .13 | 1.40 | .164 | -0.08, 0.46 |
| Perceived vaccine availability | -0.24 | 0.10 | -2.44 | .015 | -0.43, -0.05 |

*Note*. a-path: *R^2^* = .02, *F*(2,234) = 5.91, *p* = .016; b-path: *R^2^* = .02, *F*(2,233) = 1.93, *p* = .147

Total effect: *c* = 0.24, *SE* = .13, *t* = 1.78, *p* = .076, *95% CI* [-0.02, 0.51]

Indirect effect: *ab* = 0.05, *Boot* *SE* = .03, *Boot CI* [0.01, 0.12]

*N* = 238

Following these comments, I believe two minor elements of the manuscript remain inaccurate. First is the CONSORT diagram: since the manipulation check occurred *after* treatment assignment and depended on engagement with treatment, a separate box for these exclusions should occur in the branch for each treatment condition. This would clarify for reader the issue that the author and I have debated extensively. Second, the claim that “Overall, n = 98 respondents were assigned to the scarcity condition and n = 77 to the surplus condition.” on p9 is misleading. These are not the totals assigned to treatment, but the totals after applying the exclusion criteria.

**I really don’t see what I should change here.**

**With regard to the CONSORT diagram, I can just emphasize that I used the CONSORT diagram in accordance with the recommendations on the CONSORT homepage, so I don’t see where I should change it.**

**I have added every information on every sub-sample (included, excluded, condition etc.). I have also added the information on the group comparison between both experimental groups consisting in the final sample in the main manuscript and the (insignificant) differences between excluded and included sub-samples (indicating no sub-sample bias) in the supplemental materials to which I reference in the main text. I feel that it would be misleading to report the whole sub-sample comparisons in more detail in the main manuscript, as the results focus on the final sample and not the entire sample.**
